# New Andes virus isolate haplotype obtained during prospective close contacts follow-up of an Hantavirus cardiopulmonary syndrome fatal case, Chile

**DOI:** 10.1016/j.crmicr.2025.100472

**Published:** 2025-09-16

**Authors:** Aldo Barrera, Hade Ramos, Eugenia Fuentes-Luppichini, Constanza Martínez-Valdebenito, Javiera Pradenas, Catalina Rogers, Carlos Palma, Claudia Campillo, Maritza Navarrete, Nicole Le Corre, Jenniffer Angulo, Marcela Ferrés

**Affiliations:** a Departamento de Enfermedades Infecciosas e Inmunología Pediátricas, Escuela de Medicina, Pontificia Universidad Católica, Santiago, Chile; b Laboratorio de Infectología y Virología Molecular, Red Salud UC- Christus, Santiago, Chile; c Facultad de Ciencias Biológicas, Pontificia Universidad Católica, Santiago, Chile; d Seremi de Salud Región de los Ríos, Ministerio de Salud, Valdivia, Chile; e Laboratorio Biología Molecular, Hospital Base Valdivia, Chile

**Keywords:** Hantavirus, Andes virus, ANDV, Virus isolation

## Abstract

Andes virus (ANDV) is a zoonotic orthohantavirus that causes hantavirus cardiopulmonary syndrome. Transmission occurs mainly through contact with infected rodent excreta and, less frequently, between humans. Viral isolation from human samples is rare; in Chile, only one strain (CHI-7913, 2002) has been reported. The limited number of isolates reflects the challenge of obtaining samples during early viremia before antibodies appear, and maintaining long-term production of infectious particles in culture. We investigated an asymptomatic case from a family cluster, sampled during early infection. The absence of anti-ANDV antibodies in sera was confirmed by ELISA. Blood fractions were used to infect Vero E6 cells for six weeks, with infectivity monitored by RT-qPCR and Immunofluorescence. We obtained a replication-stable isolate from the buffy-coat fraction, achieving high viral loads at early passages. Whole-genome sequencing revealed a novel haplotype with mutations that reverted during non-human cell culture adaptation. This isolate, designated CHI-Hu13724, represents a new human-derived ANDV strain first reported in over two decades in Chile. It provides a valuable tool to study viral replication, infectivity, and pathogenicity.

## Highlights

• ANDV can be isolated in cell culture only in the absence of neutralizing antibodies.
• The monitoring of close contacts of ANDV cases allows to identify prospective cases.
• We isolated a new ANDV human strain from an early blood sample of a secondary case.
• CHI-Hu13724 is a different haplotype from the previous ANDV reference strains.
• Isolation of currently zoonotic ANDV strains helps the study of viral pathogenicity.

## Graphical abstract

**Figure:**
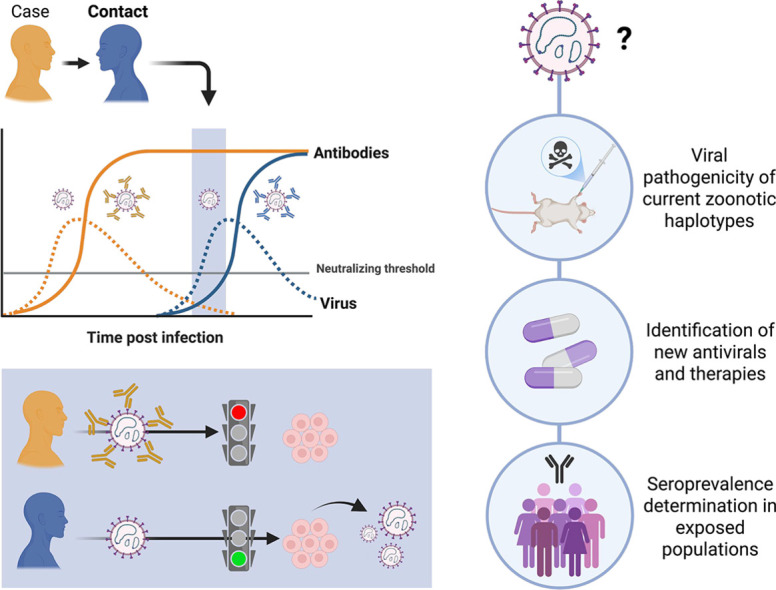

## Introduction

Hantaviruses are rodent-borne viruses belonging to the *Hantaviridae* family, which is known to cause the hantavirus cardiopulmonary syndrome (HCPS) primarily associated with New World hantaviruses, such as Sin Nombre virus (SNV) in North America, and Andes virus (ANDV) in South America (Schmaljohn and Hjelle, 1997). The ANDV, endemic to Chile and Argentina, is the only hantavirus known to be able to spread person-to-person (Martinez et al., 2005; Ferres et al., 2007; Ferres et al., 2020). Its reservoir, *Oligoryzomys longicaudatus*, is a wild rodent widely distributed in Chile (Palma et al., 2012)*.* Infection in humans ranges from asymptomatic to severe HCPS, with a lethality rate of 30–35 % (Vial et al., 2023).

ANDV is an enveloped RNA virus with three genomic segments: Large (L, encoding for the RNA-dependent RNA polymerase protein RdRp), Medium (M, encoding the glycoproteins Gn and Gc), and Small (S, encoding the nucleocapsid protein N and a non-structural protein NSs) segments (Cifuentes-Munoz et al., 2014; Vera-Otarola et al., 2012). ANDV RNA has been detected by RT-qPCR in respiratory samples, urine, blood plasma and buffy-coat, the last showing RNA detection beyond the acute phase of infection and very late in the convalescent period (Ferres et al., 2024).

The isolation of ANDV from human samples is challenging due to the short viremic interval before the rise of neutralizing antibodies that restrict viral replication (Ferres et al., 2024; Galeno et al., 2002). In Chile, only one human isolate (CHI-7913) has been reported and characterized (Galeno et al., 2002), along with one isolate from a rodent reservoir (Chile-9717869) (Meissner et al., 2002). We report the isolation and identification of a novel human-derived Chilean ANDV haplotype, CHI-Hu13724, obtained from a prospective case identified during a family cluster study.

## Results

During May to June 2024 in Región de los Ríos, Chile, a fatal hantavirus case of a woman and the consecutive infection of her husband led to clinical and virological follow-up of their two close household contacts, her grandson (case 136) and daughter (case 137). Nineteen days after the onset of symptoms of the primary case, case 136 tested positive for ANDV RNA by RT-qPCR in blood. The child, who remained under clinical follow-up, developed symptoms within 24 h, whereas his mother remained negative. Within the following three days, both subjects were transferred to a hospital where extracorporeal membrane oxygenation was available. After day 1 of admission, case 137 tested positive for ANDV and showed symptoms within 24 h. Both cases developed severe and mild diseases, respectively, and both survived.

**Fig. 1:**
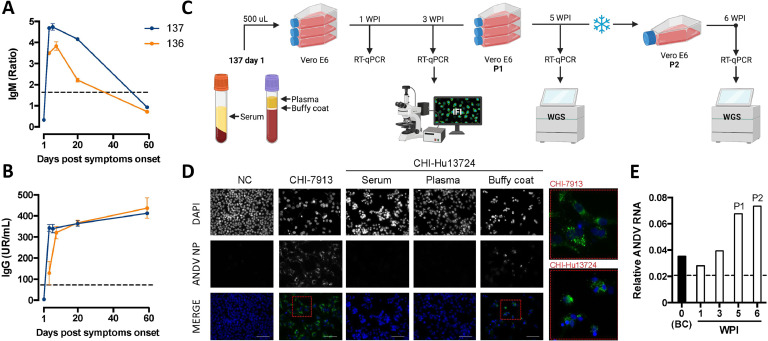
**Isolation of CHI-Hu13724 ANDV strain from a prospective case.** (A, B) Kinetics of IgM and IgG antibody response against ANDV nucleoprotein (NP) in serum samples from cases 136 and 137 monitored during the first 60 days post-symptom onset. (C) Experimental workflow used for ANDV isolation in cell culture. Serum, plasma, and buffy-coat samples collected on day 1 from case 137 were inoculated into Vero E6 cells, followed by monitoring of viral replication by RT-qPCR and indirect Immunofluorescence (IFI) up to 6 weeks post-infection (wpi). (D) Representative IFI images of Vero E6 cells showing detection of ANDV NP (green) and cell nuclei (blue, DAPI) at 3 wpi. Images include infection with the reference strain CHI-7913 (dilution 1:10,000) and uninfected negative control (NC). Red squares highlight magnified regions of merged images from CHI-7913 and CHI-Hu13724 infections. White scale bars represent 30 μm. (E) Viral RNA quantification in culture supernatants (expressed as Ct^-1^ values) at different wpi using the buffy-coat sample from case 137 as inoculum. Results are shown for viral passages 1 and 2 (P1, P2).

Serum samples from cases 136 and 137 were analyzed for anti-ANDV antibody response. Case 136 exhibited a positive IgM response four days post-symptom onset, which decreased over the following weeks, becoming undetectable after 2 months (Fig. 1A). Case 137 described a complete kinetic of IgM production and decay from day 1 to 60 post-first symptoms. Both cases maintained a sustained IgG response until sampling on day 60 post-symptom onset (Fig. 1B). Notably, case 137 showed higher IgM and IgG levels compared to case 136 at day 4 post-symptom onset (*p* < 0,005). Strikingly, the initial sample from case 137–used for blood component separation and infection assays in Vero E6 cells—showed no detectable anti-ANDV antibodies (Fig. 1B).

The viral replication in culture was monitored over six weeks under biosafety level 3 (BSL-3) laboratory conditions (Fig. 1C). At three weeks post infection (wpi), only cells inoculated with the buffy-coat tested positive for ANDV N protein detection (Fig. 1D), consistent with our previous findings that the buffy-coat fraction yields the highest ANDV detection rates (Ferres et al., 2024). A marked rise in ANDV RNA levels was observed in the culture supernatants at five wpi (Fig. 1E), exceeding the baseline levels from the original sample and coinciding with the first blind viral passage (P1). To establish a stable viral stock, P1 culture supernatants were stored at -80 °C, thawed and re-cultured, obtaining a second viral passage (P2) with a similar ANDV RNA level.

To characterize the genetic profile of the isolate named CHI-Hu13724, we performed whole-genome sequencing on the original samples from cases 136, 137, and passages P1 and P2 of 137. Genome sequencing yielded high-quality data for all samples and showed larger genome coverage than the previously reported reference CHI-7913 (Genbank accession numbers, S: MT956618, M: MT956619, L: MT956620). All segments, S, M, and L had mean depths over 7.000x (Suppl. Table 1). These genomes were compared with previously reported human strains as reference, including CHI-7913 and a representative Argentinian strain from the 2018–2019 Epuyén outbreak (Martinez et al., 2020) (Table 1). Pairwise comparisons revealed that the complete ANDV genome from case 137 is 100 % identical to case 136, consistent with their epidemiologic link. Both cases sequences and CHI-Hu13724 P1 isolate sequence shared 96.67 % nucleotide identity with CHI-7913, with a segment-specific divergence of S: 2.53 %, M: 3.37 %, and L: 3.53 % (Table 1, Suppl. Table 2). A total of 12 amino acid substitutions were identified across the three segments. However, CHI-Hu13724 P2 showed two reverting mutations (N:N46S, and NSs:I20V) and resulting in lower divergence from CHI-7913. In contrast, we observed that CHI-Hu13724 was more divergent from the Epuyén strain with a greater number of amino acid differences (*n* = 19), particularly in the M and L segments.

**Table 1:** Comparative nucleotide divergence and amino acid variation of CHI-Hu13724 with ANDV human reference sequences.

			**Case 137**	**CHI-Hu13724 P1**	**CHI-Hu13724 P2**
Compared to CHI-7913	S (1859 bp)	ND* (%)	2.53	2.53	2.47
		N	N46S	N46S	–
		NSs	I20V, S35L, D37G	I20V, S35L, D37G	S35L, D37G
	M (3654 bp)	ND* (%)	3.37	3.37	3.37
		Signal peptide	I11V	I11V	I11V
		Gn	I96V	I96V	I96V
		Gc	A920T, S1037A, V1109I	A920T, S1037A, V1109I	A920T, S1037A, V1109I
	L (6549 bp)	ND* (%)	3.53	3.53	3.53
		RdRp	R144K, A541V, A581S	R144K, A541V, A581S	R144K, A541V, A581S
Compared to Epuyén/18–19 Patient 1	S (1859 bp)	ND* (%)	4.05	4.05	4.1
		N	–	–	S46N
		NSs	S35L, R40Q, S47N	S35L, R40Q, S47N	V20I, S35L, R40Q, S47N
	M (3654 bp)	ND* (%)	4.44	4.44	4.44
		Gn	I114V, L216F, I353V, I499V	I114V, L216F, I353V, I499V	I114V, L216F, I353V, I499V
		Gc	I641T, A938T, S1055A, I1115V	I641T, A938T, S1055A, I1115V	I641T, A938T, S1055A, I1115V
	L (6549 bp)	ND* (%)	4.68	4.68	4.68
		RdRp	V141I, R144K, S364N, I402V, A541V, A581S, A876S, N1440S, V1665I, H1965Q, T2113A	V141I, R144K, S364N, I402V, A541V, A581S, A876S, N1440S, V1665I, H1965Q, T2113A	V141I, R144K, S364N, I402V, A541V, A581S, A876S, N1440S, V1665I, H1965Q, T2113A

⁎ ND = Nucleotide distance.

**Fig. 2:**
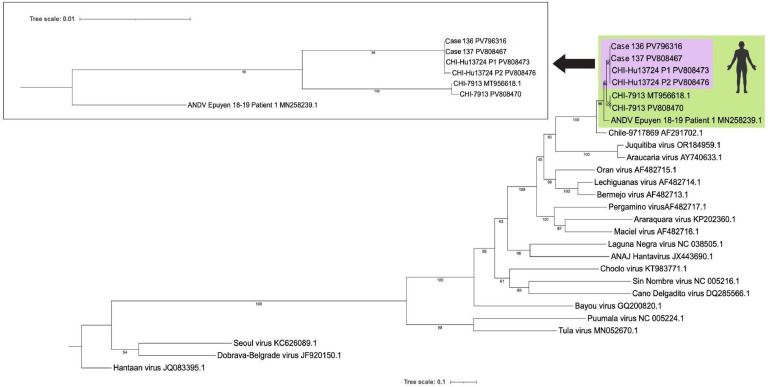
**Phylogenetic analysis of the CHI-Hu13724 ANDV isolate based on the S segment.** Maximum likelihood phylogenetic tree constructed with full-length S segment sequences (∼1.859 bp) from multiple ANDV strains and other hantaviruses. Sequences obtained from cases 136 and 137, and the CHI-Hu13724 isolate (viral passages P1 and P2) are highlighted in light purple. Additional human ANDV sequences are highlighted in greed, including the Chilean strain CHI-7913 and the Argentine Epuyén 2018–2019 patient 1. A magnified view of the relevant ANDV clade is shown in the inset (upper left). Bootstrap support values above 60 % are indicated at key nodes. The scale bar represents the number of nucleotide substitutions per site. Sequence identifiers correspond to GenBank accession codes.

Phylogenetic analysis of the S, M, and L sequences confirmed that the four reported sequences formed a well-supported distinct clade, clearly separated from CHI-7913 reference strain and the Argentinian strain (Epuyén 2018-19 patient 1) (Fig. 2, Suppl. Fig. 1). This branching pattern and high bootstrap values support the classification of CHI-Hu13724 as a novel human-derived ANDV haplotype circulating in Chile.

## Discussion

Virus isolation is one of the most reliable sources of evidence of infection and is still considered a gold standard for establishing the viral etiology of a disease. The isolation of a new haplotype provides a critical tool for enhancing diagnostic accuracy, assessing antivirals, and exploring vaccine candidates. Here we report the successful isolation of a novel ANDV haplotype, achieved after 20 years since the previously reported human isolate in Chile, using samples obtained from viremic and asymptomatic cases prior to the acute phase of the disease. For ANDV, the viremic phase lasts for almost a month after symptom onset (Ferres et al., 2024); however, the period during which infectious particles are present without specific antibodies is very brief, providing an opportunity to successfully obtain a culture-stable isolate. Our findings are consistent with the evidence reported by Galeno et al. 2002, who described the first Chilean isolate (CHI-7913) under similar conditions (Galeno et al., 2002).

The genomic and phylogenetic analysis of the CHI-Hu13724 isolate provides novel insights into the current genetic landscape of Andes virus (ANDV) in Chile. Cases-derived sequences and the CHI-Hu13724 P1 isolate sequence shared 96.67 % nucleotide identity with CHI-7913, with segment-specific divergences of S: 2.53 %, M: 3.37 %, and L: 3.53 % (Table 1). Across the three genomic segments, we identified 12 amino acid substitutions, most of which were maintained in CHI-Hu13724 P1. In contrast, CHI-Hu13724 P2 displayed two reversion mutations (N:N46S and NSs:I20V), resulting in reduced divergence from CHI-7913 and likely reflecting early adaptations to monkey cell culture (Bellomo et al., 2023). Notably, the NSs:I20V (also present in the Argentinian S sequence) might be involved in modulating the type I interferon pathway, while the N:N46S could affect N-RdRp binding and viral particle assembly (Cheng et al., 2014; Vera-Otarola et al., 2020). Interestingly, Bellomo et al. (2023) reported the same substitution in a rodent-derived ANDV strain: their isolate initially carried S at this position, but after nine successive passages in cell culture, the N variant emerged and became fixed. This convergence suggests that N46S may represent an adaptive change relevant to both *in vitro* and *in vivo* contexts.

Phylogenetic analysis of the S, M, and L segments confirmed that the four reported sequences cluster in a well-supported clade, clearly distinct from CHI-7913 and Argentinean variants such as Lechiguanas and Orán, with Epuyén being the closest but still divergent (Fig. 2, Suppl. Fig. 1). This pattern supports the classification of CHI-Hu13724 as a novel human-derived ANDV haplotype circulating in Chile. Although our dataset does not allow detailed resolution of intra-Chilean diversity, a previous work by Medina et al. (2009), based on partial S-segment sequences, suggested the existence of at least two distinct clades in Chile. Together with our results, this raises the possibility that regional ecological or epidemiological factors may be shaping locally evolving lineages, although more complete genome data are needed to confirm these trends. While CHI-7913 has long served as the reference genome, its extensive passage history in cell culture may have introduced mutations unrelated to natural transmission. In contrast, CHI-Hu13724 retains novel mutations directly sampled during acute human infection, providing a more accurate representation of circulating strains. Altogether, these findings highlight CHI-Hu13724 as a valuable resource to investigate viral determinants of transmissibility, pathogenicity, and immune evasion.

In summary, our genomic and phylogenetic analyses point to the existence of geographically distinct, evolving ANDV lineages, emphasizing the value of sequencing recent cases-derived isolates. CHI-Hu13724 represents a key reference for exploring viral determinants of transmissibility, pathogenicity, and immune evasion, while underscoring the need for continuous genomic surveillance to track hantavirus diversity and dynamics in South America. From a public health perspective, these findings underscore the importance of close epidemiological surveillance of secondary cases, whether arising from environmental exposure or person-to-person transmission.

## Materials and methods

### Anti-ANDV antibody determination

Whole-blood samples were centrifuged to separate blood fractions, and serum fractions were diluted 1:100 and used to measure anti-ANDV IgM and IgG antibodies using the ELISA Anti-Hantavirus Pool 2 América kits (#EI 278h-9601-2M and -2G, respectively, Euroimmun) according to the manufacturer’s protocols. The IgM levels were expressed as the optical density (OD) ratio of the sample and the kit calibrator, with an OD ratio ≥1.1 considered as positive, while the IgG levels were expressed as relative units (RU) per mL of sample calculated by a calibrator standard curve included in the IgG kit, with a limit of quantification of 22 RU/mL.

### Viral isolation

The isolation protocol followed Galeno et al. (2002). Serum, plasma, and buffy-coat samples from case 137, day 1 post-symptom onset, were used to inoculate Vero E6 cells at 80 % confluence in T25 flasks. Samples (500 µL) were diluted 1:2 in DMEM with 2 % heat-inactivated FBS and incubated 1 h at 37 °C with gentle agitation. Then, 5 mL of supplemented media (10 % FBS, sodium pyruvate, non-essential amino acids, Pen-Strep-Neo, and amphotericin B) was added. Cultures were maintained for 3 weeks at 37 °C, 5 % CO₂, with weekly refeeding. A blind passage was performed at 3 wpi using culture supernatants. At 5 wpi, supernatants were aliquoted (P1) and stored at –80 °C. After 48 h, a P1 aliquot was used to infect fresh cells in T175 flasks. After 1 week, new supernatants were collected (P2) and stored.

### Immunofluorescence assay

After 3 weeks post-infection, Vero E6 cells were trypsinized, washed and cultured on coverslips, and maintained in culture medium for 48 h. Cells were then fixed with 4 % paraformaldehyde overnight at 4 °C, permeabilized with PBS 0,03 % Triton X-100, and stained with a specific mouse primary anti-ANDV Nucleoprotein antibody (Barriga et al., 2013), kindly provided by Dr. Nicole Tischler, diluted 1:1000, followed by a goat anti-mouse IgG Alexa Fluor 488 secondary antibody (A11001, ThermoFisher) diluted 1:5000, and DAPI (D1306, ThermoFisher) diluted 1:1000.

### Viral RNA detection by RT-qPCR

Total nucleic acids from buffy-coat and supernatant samples were extracted using magLEAD® 12gC system (#12C2202B0753 Precision System Science Co.) following the manufacturer's protocol. To detect viral RNA, a specific real-time RT-qPCR assay (#06754155001, Roche Diagnostic GmbH) targeting a conserved region of the S segment of ANDV used routinely in clinical diagnostics (Vial et al., 2016; Markotic, 2024).

### Amplification of ANDV RNA segments and whole genome sequencing

Total RNA was extracted from 200 µL of culture supernatants using a magLEAD® 12gC system, following the manufacturer's instructions. The amplification of the S, M, and L segments of ANDV was performed as described by Taylor et al. (2021). Amplicons were quantified with the Qubit™ dsDNA HS Assay Kit on a Qubit™ 4 fluorometer. Libraries from the independent ANDV amplified DNA amplicons were prepared using the Nextera XT DNA Library Prep kit (#FC-131-1096, Illumina) and Index kit (#FC-131-1001, Illumina) following the manufacturer’s instructions. Library quality was assessed, pooled, and sequenced on the iSeq™ 100 system using a paired-end strategy (#20021532) and the iSeq™ 100 i1 Reagent v2 kit (#20031371, Illumina).

### Bioinformatic analysis

FASTQ files were downloaded from Illumina BaseSpace and processed on the Galaxy web server. Quality was assessed with FastQC and Fasta Statistics tools. Adapters and low-quality bases were trimmed using Trimmomatic (Bolger et al., 2014), applying the IlluminaClip and SlidingWindow methods. Processed reads were aligned using BWA-MEM, with the Chile-9717869 genome as the reference. Finally, consensus sequences were generated using ivar consensus (*Q* > 30, depth >400x) and aligned with Clustal Omega. Phylogenetic trees were constructed using IQ-TREE (Nguyen et al., 2015), with ModelFinder for model selection. The best-fit model according to BIC was TVM+*F* + *I* + R3 for the small segment and GTR+*F* + *I* + R3 for the medium and large segments. Bootstrap support was based on 1000 maximum-likelihood replicates. Sequencing data have been deposited in GenBank, under the following accesion numbers in order per segment (S, M, L): CHI-7913 (PV808470, PV808471, PV808472); Case 136 (PV796316, PV796317, PV796318); Case 137 (PV808467, PV808468, PV808469); CHI-Hu13724 P1 (PV808473, PV808474, PV808475); and CHI-Hu13724 P2 (PV808476, PV808477, PV808478).

## Disclaimers

The opinions expressed by authors contributing to this journal do not necessarily reflect the opinions of funders or Pontificia Universidad Católica, or Ministerio de Salud Chile. The authors declare no financial interest or conflicts of interest. The funders had no role in study design, data collection and analysis, decision to publish, or preparation of the manuscript.

## CRediT authorship contribution statement

**Aldo Barrera:** Conceptualization, Methodology, Software, Validation, Formal analysis, Investigation, Data curation, Writing – original draft, Visualization. **Hade Ramos:** Methodology, Software, Formal analysis, Investigation, Data curation, Writing – original draft, Visualization. **Eugenia Fuentes-Luppichini:** Methodology, Investigation. **Constanza Martínez-Valdebenito:** Conceptualization, Methodology. **Javiera Pradenas:** Investigation, Data curation. **Catalina Rogers:** Investigation, Data curation. **Carlos Palma:** Investigation. **Claudia Campillo:** Investigation. **Maritza Navarrete:** Investigation. **Nicole Le Corre:** Investigation. **Jenniffer Angulo:** Conceptualization, Project administration, Funding acquisition. **Marcela Ferrés:** Conceptualization, Methodology, Validation, Resources, Writing – original draft, Supervision, Project administration, Funding acquisition.

## Declaration of competing interest

The authors declare that they have no known competing financial interests or personal relationships that could have appeared to influence the work reported in this paper.

## Data Availability

Data will be made available on request.

## Appendix Supplementary materials

Image, application 1
