# Supplementary material for: New Andes virus isolate haplotype obtained during prospective close contacts follow-up of an Hantavirus cardiopulmonary syndrome fatal case, Chile
Source: Curr Res Microb Sci. 2025 Sep 16;9:100472. doi: 10.1016/j.crmicr.2025.100472 (PMC12506574; doi:10.1016/j.crmicr.2025.100472)
Supplement: Supplementary file 1 [file mmc1.zip › CRMS_SuppTable1.pdf]

Sample	Small Segment		Medium Segment		Large Segment	
	Average depth of coverage	% Genome coverage	Average depth of coverage	% Genome coverage	Average depth of coverage	% Genome coverage
CHI-7913	11,120	100%	13,178	100%	12,871	100%
Patient 136	5,979	100%	5,593	100%	5,963	100%
Patient 137	4,962	100%	4,056	100%	5,522	100%
CHI-Hu13724 P1	11,805	100%	11,621	100%	12,317	100%
CHI-Hu13724 P2	3,876	100%	4,184	100%	2,594	100%
**Average**	**7,548**	**100%**	**7,726**	**100%**	**7,853**	**100%**

* The percentage of genome coverage was compared to the CHI-7913.
