# Supplementary material for: New Andes virus isolate haplotype obtained during prospective close contacts follow-up of an Hantavirus cardiopulmonary syndrome fatal case, Chile
Source: Curr Res Microb Sci. 2025 Sep 16;9:100472. doi: 10.1016/j.crmicr.2025.100472 (PMC12506574; doi:10.1016/j.crmicr.2025.100472)
Supplement: Supplementary file 1 [file mmc1.zip › CRMS_SuppTable2.pdf]

Compared to CHI-7913						
**Segment**	**Position (nt)**	**REF**	**ALT**	**Change N**	**/NSs (+1)**	**Samples with mutation**
**S**	177	A	G	N46S	I20V	CHI-Hu13724_P1, Case_136, Case_137
**S**	200	T	C			CHI-Hu13724_P1, CHI-Hu13724_P2, Case_136, Case_137
**S**	223	C	T		S35L	CHI-Hu13724_P1, CHI-Hu13724_P2, Case_136, Case_137
**S**	229	A	G		D37G	CHI-Hu13724_P1, CHI-Hu13724_P2, Case_136, Case_137
**S**	326	T	C			CHI-Hu13724_P1, CHI-Hu13724_P2, Case_136, Case_137
**S**	352	T	C			CHI-Hu13724_P1, CHI-Hu13724_P2, Case_136, Case_137
**S**	361	C	T			CHI-Hu13724_P1, CHI-Hu13724_P2, Case_136, Case_137
**S**	365	T	C			CHI-Hu13724_P1, CHI-Hu13724_P2, Case_136, Case_137
**S**	376	G	A			CHI-Hu13724_P1, CHI-Hu13724_P2, Case_136, Case_137
**S**	451	C	T			CHI-Hu13724_P1, CHI-Hu13724_P2, Case_136, Case_137
**S**	463	G	A			CHI-Hu13724_P1, CHI-Hu13724_P2, Case_136, Case_137
**S**	475	A	G			CHI-Hu13724_P1, CHI-Hu13724_P2, Case_136, Case_137
**S**	559	A	G			CHI-Hu13724_P1, CHI-Hu13724_P2, Case_136, Case_137
**S**	562	G	A			CHI-Hu13724_P1, CHI-Hu13724_P2, Case_136, Case_137
**S**	613	G	A			CHI-Hu13724_P1, CHI-Hu13724_P2, Case_136, Case_137
**S**	619	T	C			CHI-Hu13724_P1, CHI-Hu13724_P2, Case_136, Case_137
**S**	709	C	T			CHI-Hu13724_P1, CHI-Hu13724_P2, Case_136, Case_137
**S**	727	G	A			CHI-Hu13724_P1, CHI-Hu13724_P2, Case_136, Case_137
**S**	826	G	A			CHI-Hu13724_P1, CHI-Hu13724_P2, Case_136, Case_137
**S**	835	T	C			CHI-Hu13724_P1, CHI-Hu13724_P2, Case_136, Case_137
**S**	841	T	C			CHI-Hu13724_P1, CHI-Hu13724_P2, Case_136, Case_137
**S**	856	C	T			CHI-Hu13724_P1, CHI-Hu13724_P2, Case_136, Case_137
**S**	880	C	T			CHI-Hu13724_P1, CHI-Hu13724_P2, Case_136, Case_137
**S**	910	A	G			CHI-Hu13724_P1, CHI-Hu13724_P2, Case_136, Case_137
**S**	928	A	G			CHI-Hu13724_P1, CHI-Hu13724_P2, Case_136, Case_137
**S**	970	G	A			CHI-Hu13724_P1, CHI-Hu13724_P2, Case_136, Case_137
**S**	988	G	A			CHI-Hu13724_P1, CHI-Hu13724_P2, Case_136, Case_137
**S**	1054	C	T			CHI-Hu13724_P1, CHI-Hu13724_P2, Case_136, Case_137
**S**	1081	G	A			CHI-Hu13724_P1, CHI-Hu13724_P2, Case_136, Case_137
**S**	1117	C	T			CHI-Hu13724_P1, CHI-Hu13724_P2, Case_136, Case_137
**S**	1120	T	C			CHI-Hu13724_P1, CHI-Hu13724_P2, Case_136, Case_137
**S**	1331	C	T			CHI-Hu13724_P1, CHI-Hu13724_P2, Case_136, Case_137
**S**	1334	A	G			CHI-Hu13724_P1, CHI-Hu13724_P2, Case_136, Case_137
**S**	1342	G	A			CHI-Hu13724_P1, CHI-Hu13724_P2, Case_136, Case_137
**S**	1434	T	C			CHI-Hu13724_P1, CHI-Hu13724_P2, Case_136, Case_137
**S**	1448	G	A			CHI-Hu13724_P1, CHI-Hu13724_P2, Case_136, Case_137
**S**	1487	T	G			CHI-Hu13724_P1, CHI-Hu13724_P2, Case_136, Case_137
**S**	1500	A	C			CHI-Hu13724_P1, CHI-Hu13724_P2, Case_136, Case_137
**S**	1526	C	T			CHI-Hu13724_P1, CHI-Hu13724_P2, Case_136, Case_137
**S**	1552	C	T			CHI-Hu13724_P1, CHI-Hu13724_P2, Case_136, Case_137
**S**	1576	C	T			CHI-Hu13724_P1, CHI-Hu13724_P2, Case_136, Case_137
**S**	1605	T	G			CHI-Hu13724_P1, CHI-Hu13724_P2, Case_136, Case_137
**S**	1646	C	T			CHI-Hu13724_P1, CHI-Hu13724_P2, Case_136, Case_137
**S**	1662	G	A			CHI-Hu13724_P1, CHI-Hu13724_P2, Case_136, Case_137
**S**	1667	T	A			CHI-Hu13724_P1, CHI-Hu13724_P2, Case_136, Case_137
**S**	1835	T	C			CHI-Hu13724_P1, CHI-Hu13724_P2, Case_136, Case_137
**Segment**	**Position (nt)**	**REF**	**ALT**	**Change GPC**		**Samples with mutation**
**M**	81	A	G	I11V		CHI-Hu13724_P1, CHI-Hu13724_P2, Case_136, Case_137
**M**	110	T	C			CHI-Hu13724_P1, CHI-Hu13724_P2, Case_136, Case_137
**M**	263	A	G			CHI-Hu13724_P1, CHI-Hu13724_P2, Case_136, Case_137
**M**	284	C	T			CHI-Hu13724_P1, CHI-Hu13724_P2, Case_136, Case_137
**M**	314	C	T			CHI-Hu13724_P1, CHI-Hu13724_P2, Case_136, Case_137
**M**	362	C	T			CHI-Hu13724_P1, CHI-Hu13724_P2, Case_136, Case_137
**M**	380	A	G			CHI-Hu13724_P1, CHI-Hu13724_P2, Case_136, Case_137
**M**	390	A	G	I96V		CHI-Hu13724_P1, CHI-Hu13724_P2, Case_136, Case_137
**M**	413	T	C			CHI-Hu13724_P1, CHI-Hu13724_P2, Case_136, Case_137
**M**	419	A	G			CHI-Hu13724_P1, CHI-Hu13724_P2, Case_136, Case_137
**M**	431	G	A			CHI-Hu13724_P1, CHI-Hu13724_P2, Case_136, Case_137
**M**	446	C	T			CHI-Hu13724_P1, CHI-Hu13724_P2, Case_136, Case_137
**M**	455	T	A			CHI-Hu13724_P1, CHI-Hu13724_P2, Case_136, Case_137
**M**	488	C	T			CHI-Hu13724_P1, CHI-Hu13724_P2, Case_136, Case_137
**M**	506	A	G			CHI-Hu13724_P1, CHI-Hu13724_P2, Case_136, Case_137
**M**	512	C	G			CHI-Hu13724_P1, CHI-Hu13724_P2, Case_136, Case_137
**M**	530	T	C			CHI-Hu13724_P1, CHI-Hu13724_P2, Case_136, Case_137
**M**	554	T	C			CHI-Hu13724_P1, CHI-Hu13724_P2, Case_136, Case_137
**M**	563	G	A			CHI-Hu13724_P1, CHI-Hu13724_P2, Case_136, Case_137
**M**	569	C	T			CHI-Hu13724_P1, CHI-Hu13724_P2, Case_136, Case_137

**M**	575	A	G	CHI-Hu13724_P1, CHI-Hu13724_P2, Case_136, Case_137
**M**	614	A	G	CHI-Hu13724_P1, CHI-Hu13724_P2, Case_136, Case_137
**M**	636	C	T	CHI-Hu13724_P1, CHI-Hu13724_P2, Case_136, Case_137
**M**	642	T	C	CHI-Hu13724_P1, CHI-Hu13724_P2, Case_136, Case_137
**M**	644	A	G	CHI-Hu13724_P1, CHI-Hu13724_P2, Case_136, Case_137
**M**	674	C	T	CHI-Hu13724_P1, CHI-Hu13724_P2, Case_136, Case_137
**M**	713	G	A	CHI-Hu13724_P1, CHI-Hu13724_P2, Case_136, Case_137
**M**	720	C	T	CHI-Hu13724_P1, CHI-Hu13724_P2, Case_136, Case_137
**M**	722	A	G	CHI-Hu13724_P1, CHI-Hu13724_P2, Case_136, Case_137
**M**	764	G	T	CHI-Hu13724_P1, CHI-Hu13724_P2, Case_136, Case_137
**M**	770	G	A	CHI-Hu13724_P1, CHI-Hu13724_P2, Case_136, Case_137
**M**	782	G	A	CHI-Hu13724_P1, CHI-Hu13724_P2, Case_136, Case_137
**M**	825	C	T	CHI-Hu13724_P1, CHI-Hu13724_P2, Case_136, Case_137
**M**	830	C	T	CHI-Hu13724_P1, CHI-Hu13724_P2, Case_136, Case_137
**M**	848	C	T	CHI-Hu13724_P1, CHI-Hu13724_P2, Case_136, Case_137
**M**	863	G	A	CHI-Hu13724_P1, CHI-Hu13724_P2, Case_136, Case_137
**M**	899	G	A	CHI-Hu13724_P1, CHI-Hu13724_P2, Case_136, Case_137
**M**	911	T	C	CHI-Hu13724_P1, CHI-Hu13724_P2, Case_136, Case_137
**M**	992	G	A	CHI-Hu13724_P1, CHI-Hu13724_P2, Case_136, Case_137
**M**	1013	C	T	CHI-Hu13724_P1, CHI-Hu13724_P2, Case_136, Case_137
**M**	1025	C	T	CHI-Hu13724_P1, CHI-Hu13724_P2, Case_136, Case_137
**M**	1055	A	G	CHI-Hu13724_P1, CHI-Hu13724_P2, Case_136, Case_137
**M**	1076	C	T	CHI-Hu13724_P1, CHI-Hu13724_P2, Case_136, Case_137
**M**	1178	A	G	CHI-Hu13724_P1, CHI-Hu13724_P2, Case_136, Case_137
**M**	1196	C	T	CHI-Hu13724_P1, CHI-Hu13724_P2, Case_136, Case_137
**M**	1256	C	T	CHI-Hu13724_P1, CHI-Hu13724_P2, Case_136, Case_137
**M**	1259	T	C	CHI-Hu13724_P1, CHI-Hu13724_P2, Case_136, Case_137
**M**	1307	C	T	CHI-Hu13724_P1, CHI-Hu13724_P2, Case_136, Case_137
**M**	1310	A	G	CHI-Hu13724_P1, CHI-Hu13724_P2, Case_136, Case_137
**M**	1355	C	T	CHI-Hu13724_P1, CHI-Hu13724_P2, Case_136, Case_137
**M**	1364	T	C	CHI-Hu13724_P1, CHI-Hu13724_P2, Case_136, Case_137
**M**	1370	A	G	CHI-Hu13724_P1, CHI-Hu13724_P2, Case_136, Case_137
**M**	1514	C	A	CHI-Hu13724_P1, CHI-Hu13724_P2, Case_136, Case_137
**M**	1541	A	G	CHI-Hu13724_P1, CHI-Hu13724_P2, Case_136, Case_137
**M**	1565	G	A	CHI-Hu13724_P1, CHI-Hu13724_P2, Case_136, Case_137
**M**	1593	C	T	CHI-Hu13724_P1, CHI-Hu13724_P2, Case_136, Case_137
**M**	1595	G	A	CHI-Hu13724_P1, CHI-Hu13724_P2, Case_136, Case_137
**M**	1661	C	T	CHI-Hu13724_P1, CHI-Hu13724_P2, Case_136, Case_137
**M**	1670	A	G	CHI-Hu13724_P1, CHI-Hu13724_P2, Case_136, Case_137
**M**	1718	A	G	CHI-Hu13724_P1, CHI-Hu13724_P2, Case_136, Case_137
**M**	1736	A	G	CHI-Hu13724_P1, CHI-Hu13724_P2, Case_136, Case_137
**M**	1757	C	T	CHI-Hu13724_P1, CHI-Hu13724_P2, Case_136, Case_137
**M**	1784	A	G	CHI-Hu13724_P1, CHI-Hu13724_P2, Case_136, Case_137
**M**	1826	C	T	CHI-Hu13724_P1, CHI-Hu13724_P2, Case_136, Case_137
**M**	1860	T	C	CHI-Hu13724_P1, CHI-Hu13724_P2, Case_136, Case_137
**M**	1877	G	A	CHI-Hu13724_P1, CHI-Hu13724_P2, Case_136, Case_137
**M**	1904	C	T	CHI-Hu13724_P1, CHI-Hu13724_P2, Case_136, Case_137
**M**	1928	T	C	CHI-Hu13724_P1, CHI-Hu13724_P2, Case_136, Case_137
**M**	1937	A	G	CHI-Hu13724_P1, CHI-Hu13724_P2, Case_136, Case_137
**M**	2027	T	C	CHI-Hu13724_P1, CHI-Hu13724_P2, Case_136, Case_137
**M**	2054	C	T	CHI-Hu13724_P1, CHI-Hu13724_P2, Case_136, Case_137
**M**	2081	A	G	CHI-Hu13724_P1, CHI-Hu13724_P2, Case_136, Case_137
**M**	2093	A	G	CHI-Hu13724_P1, CHI-Hu13724_P2, Case_136, Case_137
**M**	2141	A	G	CHI-Hu13724_P1, CHI-Hu13724_P2, Case_136, Case_137
**M**	2159	A	T	CHI-Hu13724_P1, CHI-Hu13724_P2, Case_136, Case_137
**M**	2162	T	C	CHI-Hu13724_P1, CHI-Hu13724_P2, Case_136, Case_137
**M**	2168	T	C	CHI-Hu13724_P1, CHI-Hu13724_P2, Case_136, Case_137
**M**	2171	T	C	CHI-Hu13724_P1, CHI-Hu13724_P2, Case_136, Case_137
**M**	2246	C	T	CHI-Hu13724_P1, CHI-Hu13724_P2, Case_136, Case_137
**M**	2276	T	C	CHI-Hu13724_P1, CHI-Hu13724_P2, Case_136, Case_137
**M**	2327	T	C	CHI-Hu13724_P1, CHI-Hu13724_P2, Case_136, Case_137
**M**	2345	T	C	CHI-Hu13724_P1, CHI-Hu13724_P2, Case_136, Case_137
**M**	2387	A	G	CHI-Hu13724_P1, CHI-Hu13724_P2, Case_136, Case_137
**M**	2435	A	G	CHI-Hu13724_P1, CHI-Hu13724_P2, Case_136, Case_137
**M**	2468	A	G	CHI-Hu13724_P1, CHI-Hu13724_P2, Case_136, Case_137
**M**	2552	T	C	CHI-Hu13724_P1, CHI-Hu13724_P2, Case_136, Case_137
**M**	2555	T	C	CHI-Hu13724_P1, CHI-Hu13724_P2, Case_136, Case_137
**M**	2597	T	C	CHI-Hu13724_P1, CHI-Hu13724_P2, Case_136, Case_137

**M**	2604	C	T		CHI-Hu13724_P1, CHI-Hu13724_P2, Case_136, Case_137
**M**	2612	G	A		CHI-Hu13724_P1, CHI-Hu13724_P2, Case_136, Case_137
**M**	2613	T	C		CHI-Hu13724_P1, CHI-Hu13724_P2, Case_136, Case_137
**M**	2621	A	G		CHI-Hu13724_P1, CHI-Hu13724_P2, Case_136, Case_137
**M**	2696	T	C		CHI-Hu13724_P1, CHI-Hu13724_P2, Case_136, Case_137
**M**	2768	C	T		CHI-Hu13724_P1, CHI-Hu13724_P2, Case_136, Case_137
**M**	2774	C	T		CHI-Hu13724_P1, CHI-Hu13724_P2, Case_136, Case_137
**M**	2780	T	A		CHI-Hu13724_P1, CHI-Hu13724_P2, Case_136, Case_137
**M**	2798	T	C		CHI-Hu13724_P1, CHI-Hu13724_P2, Case_136, Case_137
**M**	2862	G	A	A920T	CHI-Hu13724_P1, CHI-Hu13724_P2, Case_136, Case_137
**M**	2894	G	A		CHI-Hu13724_P1, CHI-Hu13724_P2, Case_136, Case_137
**M**	2897	T	C		CHI-Hu13724_P1, CHI-Hu13724_P2, Case_136, Case_137
**M**	2912	A	C		CHI-Hu13724_P1, CHI-Hu13724_P2, Case_136, Case_137
**M**	2963	C	T		CHI-Hu13724_P1, CHI-Hu13724_P2, Case_136, Case_137
**M**	3029	T	C		CHI-Hu13724_P1, CHI-Hu13724_P2, Case_136, Case_137
**M**	3041	C	T		CHI-Hu13724_P1, CHI-Hu13724_P2, Case_136, Case_137
**M**	3053	G	A		CHI-Hu13724_P1, CHI-Hu13724_P2, Case_136, Case_137
**M**	3116	A	G		CHI-Hu13724_P1, CHI-Hu13724_P2, Case_136, Case_137
**M**	3122	C	T		CHI-Hu13724_P1, CHI-Hu13724_P2, Case_136, Case_137
**M**	3128	T	C		CHI-Hu13724_P1, CHI-Hu13724_P2, Case_136, Case_137
**M**	3146	A	G		CHI-Hu13724_P1, CHI-Hu13724_P2, Case_136, Case_137
**M**	3149	A	G		CHI-Hu13724_P1, CHI-Hu13724_P2, Case_136, Case_137
**M**	3158	T	C		CHI-Hu13724_P1, CHI-Hu13724_P2, Case_136, Case_137
**M**	3209	C	T		CHI-Hu13724_P1, CHI-Hu13724_P2, Case_136, Case_137
**M**	3212	C	T		CHI-Hu13724_P1, CHI-Hu13724_P2, Case_136, Case_137
**M**	3213	T	G	S1037A	CHI-Hu13724_P1, CHI-Hu13724_P2, Case_136, Case_137
**M**	3242	C	T		CHI-Hu13724_P1, CHI-Hu13724_P2, Case_136, Case_137
**M**	3429	G	A	V1109I	CHI-Hu13724_P1, CHI-Hu13724_P2, Case_136, Case_137
**M**	3467	G	A		CHI-Hu13724_P1, CHI-Hu13724_P2, Case_136, Case_137
**M**	3476	C	T		CHI-Hu13724_P1, CHI-Hu13724_P2, Case_136, Case_137
**M**	3481	A	G		CHI-Hu13724_P1, CHI-Hu13724_P2, Case_136, Case_137
**M**	3485	C	T		CHI-Hu13724_P1, CHI-Hu13724_P2, Case_136, Case_137
**M**	3489	T	A		CHI-Hu13724_P1, CHI-Hu13724_P2, Case_136, Case_137
**M**	3515	C	T		CHI-Hu13724_P1, CHI-Hu13724_P2, Case_136, Case_137
**M**	3516	C	T		CHI-Hu13724_P1, CHI-Hu13724_P2, Case_136, Case_137

Segment	Position (nt)	REF	ALT	Change RdRp	Samples with mutation
**L**	136	C	T		CHI-Hu13724_P1, CHI-Hu13724_P2, Case_136, Case_137
**L**	146	C	T		CHI-Hu13724_P1, CHI-Hu13724_P2, Case_136, Case_137
**L**	181	T	C		CHI-Hu13724_P1, CHI-Hu13724_P2, Case_136, Case_137
**L**	214	T	C		CHI-Hu13724_P1, CHI-Hu13724_P2, Case_136, Case_137
**L**	215	C	T		CHI-Hu13724_P1, CHI-Hu13724_P2, Case_136, Case_137
**L**	232	A	C		CHI-Hu13724_P1, CHI-Hu13724_P2, Case_136, Case_137
**L**	313	G	A		CHI-Hu13724_P1, CHI-Hu13724_P2, Case_136, Case_137
**L**	358	T	C		CHI-Hu13724_P1, CHI-Hu13724_P2, Case_136, Case_137
**L**	388	G	A		CHI-Hu13724_P1, CHI-Hu13724_P2, Case_136, Case_137
**L**	406	A	G		CHI-Hu13724_P1, CHI-Hu13724_P2, Case_136, Case_137
**L**	427	C	T		CHI-Hu13724_P1, CHI-Hu13724_P2, Case_136, Case_137
**L**	433	A	G		CHI-Hu13724_P1, CHI-Hu13724_P2, Case_136, Case_137
**L**	451	T	C		CHI-Hu13724_P1, CHI-Hu13724_P2, Case_136, Case_137
**L**	465	G	A	R144K	CHI-Hu13724_P1, CHI-Hu13724_P2, Case_136, Case_137
**L**	481	A	G		CHI-Hu13724_P1, CHI-Hu13724_P2, Case_136, Case_137
**L**	487	C	T		CHI-Hu13724_P1, CHI-Hu13724_P2, Case_136, Case_137
**L**	505	G	A		CHI-Hu13724_P1, CHI-Hu13724_P2, Case_136, Case_137
**L**	538	C	T		CHI-Hu13724_P1, CHI-Hu13724_P2, Case_136, Case_137
**L**	544	C	T		CHI-Hu13724_P1, CHI-Hu13724_P2, Case_136, Case_137
**L**	559	T	G		CHI-Hu13724_P1, CHI-Hu13724_P2, Case_136, Case_137
**L**	565	T	C		CHI-Hu13724_P1, CHI-Hu13724_P2, Case_136, Case_137
**L**	617	A	C		CHI-Hu13724_P1, CHI-Hu13724_P2, Case_136, Case_137
**L**	673	A	G		CHI-Hu13724_P1, CHI-Hu13724_P2, Case_136, Case_137
**L**	712	C	T		CHI-Hu13724_P1, CHI-Hu13724_P2, Case_136, Case_137
**L**	763	G	A		CHI-Hu13724_P1, CHI-Hu13724_P2, Case_136, Case_137
**L**	775	T	C		CHI-Hu13724_P1, CHI-Hu13724_P2, Case_136, Case_137
**L**	778	A	G		CHI-Hu13724_P1, CHI-Hu13724_P2, Case_136, Case_137
**L**	820	T	C		CHI-Hu13724_P1, CHI-Hu13724_P2, Case_136, Case_137
**L**	835	A	G		CHI-Hu13724_P1, CHI-Hu13724_P2, Case_136, Case_137
**L**	850	A	G		CHI-Hu13724_P1, CHI-Hu13724_P2, Case_136, Case_137
**L**	919	G	A		CHI-Hu13724_P1, CHI-Hu13724_P2, Case_136, Case_137
**L**	934	G	A		CHI-Hu13724_P1, CHI-Hu13724_P2, Case_136, Case_137

**L**	1060	A	G		CHI-Hu13724_P1, CHI-Hu13724_P2, Case_136, Case_137
**L**	1066	T	C		CHI-Hu13724_P1, CHI-Hu13724_P2, Case_136, Case_137
**L**	1087	C	T		CHI-Hu13724_P1, CHI-Hu13724_P2, Case_136, Case_137
**L**	1093	C	T		CHI-Hu13724_P1, CHI-Hu13724_P2, Case_136, Case_137
**L**	1096	T	C		CHI-Hu13724_P1, CHI-Hu13724_P2, Case_136, Case_137
**L**	1147	A	G		CHI-Hu13724_P1, CHI-Hu13724_P2, Case_136, Case_137
**L**	1228	A	G		CHI-Hu13724_P1, CHI-Hu13724_P2, Case_136, Case_137
**L**	1273	G	A		CHI-Hu13724_P1, CHI-Hu13724_P2, Case_136, Case_137
**L**	1306	C	T		CHI-Hu13724_P1, CHI-Hu13724_P2, Case_136, Case_137
**L**	1309	A	G		CHI-Hu13724_P1, CHI-Hu13724_P2, Case_136, Case_137
**L**	1327	C	T		CHI-Hu13724_P1, CHI-Hu13724_P2, Case_136, Case_137
**L**	1378	A	G		CHI-Hu13724_P1, CHI-Hu13724_P2, Case_136, Case_137
**L**	1381	T	C		CHI-Hu13724_P1, CHI-Hu13724_P2, Case_136, Case_137
**L**	1384	T	C		CHI-Hu13724_P1, CHI-Hu13724_P2, Case_136, Case_137
**L**	1387	A	G		CHI-Hu13724_P1, CHI-Hu13724_P2, Case_136, Case_137
**L**	1399	C	T		CHI-Hu13724_P1, CHI-Hu13724_P2, Case_136, Case_137
**L**	1423	C	T		CHI-Hu13724_P1, CHI-Hu13724_P2, Case_136, Case_137
**L**	1432	T	C		CHI-Hu13724_P1, CHI-Hu13724_P2, Case_136, Case_137
**L**	1435	T	C		CHI-Hu13724_P1, CHI-Hu13724_P2, Case_136, Case_137
**L**	1438	A	G		CHI-Hu13724_P1, CHI-Hu13724_P2, Case_136, Case_137
**L**	1465	T	C		CHI-Hu13724_P1, CHI-Hu13724_P2, Case_136, Case_137
**L**	1474	T	C		CHI-Hu13724_P1, CHI-Hu13724_P2, Case_136, Case_137
**L**	1615	G	A		CHI-Hu13724_P1, CHI-Hu13724_P2, Case_136, Case_137
**L**	1656	C	T	A541V	CHI-Hu13724_P1, CHI-Hu13724_P2, Case_136, Case_137
**L**	1657	A	G		CHI-Hu13724_P1, CHI-Hu13724_P2, Case_136, Case_137
**L**	1660	C	T		CHI-Hu13724_P1, CHI-Hu13724_P2, Case_136, Case_137
**L**	1705	T	C		CHI-Hu13724_P1, CHI-Hu13724_P2, Case_136, Case_137
**L**	1735	G	A		CHI-Hu13724_P1, CHI-Hu13724_P2, Case_136, Case_137
**L**	1744	A	C		CHI-Hu13724_P1, CHI-Hu13724_P2, Case_136, Case_137
**L**	1775	G	T	A581S	CHI-Hu13724_P1, CHI-Hu13724_P2, Case_136, Case_137
**L**	1780	A	G		CHI-Hu13724_P1, CHI-Hu13724_P2, Case_136, Case_137
**L**	1792	C	T		CHI-Hu13724_P1, CHI-Hu13724_P2, Case_136, Case_137
**L**	1840	A	G		CHI-Hu13724_P1, CHI-Hu13724_P2, Case_136, Case_137
**L**	1948	C	T		CHI-Hu13724_P1, CHI-Hu13724_P2, Case_136, Case_137
**L**	1963	G	A		CHI-Hu13724_P1, CHI-Hu13724_P2, Case_136, Case_137
**L**	1984	A	G		CHI-Hu13724_P1, CHI-Hu13724_P2, Case_136, Case_137
**L**	1990	A	T		CHI-Hu13724_P1, CHI-Hu13724_P2, Case_136, Case_137
**L**	2056	G	A		CHI-Hu13724_P1, CHI-Hu13724_P2, Case_136, Case_137
**L**	2119	G	T		CHI-Hu13724_P1, CHI-Hu13724_P2, Case_136, Case_137
**L**	2131	C	T		CHI-Hu13724_P1, CHI-Hu13724_P2, Case_136, Case_137
**L**	2182	T	C		CHI-Hu13724_P1, CHI-Hu13724_P2, Case_136, Case_137
**L**	2209	T	C		CHI-Hu13724_P1, CHI-Hu13724_P2, Case_136, Case_137
**L**	2221	G	A		CHI-Hu13724_P1, CHI-Hu13724_P2, Case_136, Case_137
**L**	2242	A	G		CHI-Hu13724_P1, CHI-Hu13724_P2, Case_136, Case_137
**L**	2245	A	G		CHI-Hu13724_P1, CHI-Hu13724_P2, Case_136, Case_137
**L**	2251	A	G		CHI-Hu13724_P1, CHI-Hu13724_P2, Case_136, Case_137
**L**	2275	T	C		CHI-Hu13724_P1, CHI-Hu13724_P2, Case_136, Case_137
**L**	2290	A	G		CHI-Hu13724_P1, CHI-Hu13724_P2, Case_136, Case_137
**L**	2327	C	T		CHI-Hu13724_P1, CHI-Hu13724_P2, Case_136, Case_137
**L**	2335	A	G		CHI-Hu13724_P1, CHI-Hu13724_P2, Case_136, Case_137
**L**	2359	C	T		CHI-Hu13724_P1, CHI-Hu13724_P2, Case_136, Case_137
**L**	2362	T	C		CHI-Hu13724_P1, CHI-Hu13724_P2, Case_136, Case_137
**L**	2389	C	T		CHI-Hu13724_P1, CHI-Hu13724_P2, Case_136, Case_137
**L**	2401	G	A		CHI-Hu13724_P1, CHI-Hu13724_P2, Case_136, Case_137
**L**	2404	T	G		CHI-Hu13724_P1, CHI-Hu13724_P2, Case_136, Case_137
**L**	2407	A	G		CHI-Hu13724_P1, CHI-Hu13724_P2, Case_136, Case_137
**L**	2446	G	A		CHI-Hu13724_P1, CHI-Hu13724_P2, Case_136, Case_137
**L**	2497	G	A		CHI-Hu13724_P1, CHI-Hu13724_P2, Case_136, Case_137
**L**	2546	C	T		CHI-Hu13724_P1, CHI-Hu13724_P2, Case_136, Case_137
**L**	2548	T	G		CHI-Hu13724_P1, CHI-Hu13724_P2, Case_136, Case_137
**L**	2578	A	G		CHI-Hu13724_P1, CHI-Hu13724_P2, Case_136, Case_137
**L**	2611	A	G		CHI-Hu13724_P1, CHI-Hu13724_P2, Case_136, Case_137
**L**	2620	T	C		CHI-Hu13724_P1, CHI-Hu13724_P2, Case_136, Case_137
**L**	2650	G	A		CHI-Hu13724_P1, CHI-Hu13724_P2, Case_136, Case_137
**L**	2653	C	T		CHI-Hu13724_P1, CHI-Hu13724_P2, Case_136, Case_137
**L**	2671	G	A		CHI-Hu13724_P1, CHI-Hu13724_P2, Case_136, Case_137
**L**	2689	A	G		CHI-Hu13724_P1, CHI-Hu13724_P2, Case_136, Case_137
**L**	2719	T	C		CHI-Hu13724_P1, CHI-Hu13724_P2, Case_136, Case_137

**L**	2725	T	C	CHI-Hu13724_P1, CHI-Hu13724_P2, Case_136, Case_137
**L**	2752	G	A	CHI-Hu13724_P1, CHI-Hu13724_P2, Case_136, Case_137
**L**	2773	C	T	CHI-Hu13724_P1, CHI-Hu13724_P2, Case_136, Case_137
**L**	2779	A	G	CHI-Hu13724_P1, CHI-Hu13724_P2, Case_136, Case_137
**L**	2782	T	C	CHI-Hu13724_P1, CHI-Hu13724_P2, Case_136, Case_137
**L**	2803	G	A	CHI-Hu13724_P1, CHI-Hu13724_P2, Case_136, Case_137
**L**	2857	G	A	CHI-Hu13724_P1, CHI-Hu13724_P2, Case_136, Case_137
**L**	2917	C	T	CHI-Hu13724_P1, CHI-Hu13724_P2, Case_136, Case_137
**L**	2929	A	G	CHI-Hu13724_P1, CHI-Hu13724_P2, Case_136, Case_137
**L**	2980	C	T	CHI-Hu13724_P1, CHI-Hu13724_P2, Case_136, Case_137
**L**	2986	C	T	CHI-Hu13724_P1, CHI-Hu13724_P2, Case_136, Case_137
**L**	3023	C	T	CHI-Hu13724_P1, CHI-Hu13724_P2, Case_136, Case_137
**L**	3031	C	T	CHI-Hu13724_P1, CHI-Hu13724_P2, Case_136, Case_137
**L**	3038	C	T	CHI-Hu13724_P1, CHI-Hu13724_P2, Case_136, Case_137
**L**	3187	C	T	CHI-Hu13724_P1, CHI-Hu13724_P2, Case_136, Case_137
**L**	3190	T	C	CHI-Hu13724_P1, CHI-Hu13724_P2, Case_136, Case_137
**L**	3220	G	A	CHI-Hu13724_P1, CHI-Hu13724_P2, Case_136, Case_137
**L**	3235	G	A	CHI-Hu13724_P1, CHI-Hu13724_P2, Case_136, Case_137
**L**	3265	C	T	CHI-Hu13724_P1, CHI-Hu13724_P2, Case_136, Case_137
**L**	3271	G	A	CHI-Hu13724_P1, CHI-Hu13724_P2, Case_136, Case_137
**L**	3397	A	G	CHI-Hu13724_P1, CHI-Hu13724_P2, Case_136, Case_137
**L**	3421	C	T	CHI-Hu13724_P1, CHI-Hu13724_P2, Case_136, Case_137
**L**	3457	A	G	CHI-Hu13724_P1, CHI-Hu13724_P2, Case_136, Case_137
**L**	3508	A	G	CHI-Hu13724_P1, CHI-Hu13724_P2, Case_136, Case_137
**L**	3529	A	T	CHI-Hu13724_P1, CHI-Hu13724_P2, Case_136, Case_137
**L**	3535	C	T	CHI-Hu13724_P1, CHI-Hu13724_P2, Case_136, Case_137
**L**	3559	T	C	CHI-Hu13724_P1, CHI-Hu13724_P2, Case_136, Case_137
**L**	3605	T	C	CHI-Hu13724_P1, CHI-Hu13724_P2, Case_136, Case_137
**L**	3628	C	T	CHI-Hu13724_P1, CHI-Hu13724_P2, Case_136, Case_137
**L**	3637	T	C	CHI-Hu13724_P1, CHI-Hu13724_P2, Case_136, Case_137
**L**	3664	A	G	CHI-Hu13724_P1, CHI-Hu13724_P2, Case_136, Case_137
**L**	3691	A	G	CHI-Hu13724_P1, CHI-Hu13724_P2, Case_136, Case_137
**L**	3700	T	C	CHI-Hu13724_P1, CHI-Hu13724_P2, Case_136, Case_137
**L**	3721	T	A	CHI-Hu13724_P1, CHI-Hu13724_P2, Case_136, Case_137
**L**	3751	A	G	CHI-Hu13724_P1, CHI-Hu13724_P2, Case_136, Case_137
**L**	3880	C	T	CHI-Hu13724_P1, CHI-Hu13724_P2, Case_136, Case_137
**L**	3883	G	A	CHI-Hu13724_P1, CHI-Hu13724_P2, Case_136, Case_137
**L**	3922	C	T	CHI-Hu13724_P1, CHI-Hu13724_P2, Case_136, Case_137
**L**	3964	T	C	CHI-Hu13724_P1, CHI-Hu13724_P2, Case_136, Case_137
**L**	3994	A	G	CHI-Hu13724_P1, CHI-Hu13724_P2, Case_136, Case_137
**L**	4009	T	C	CHI-Hu13724_P1, CHI-Hu13724_P2, Case_136, Case_137
**L**	4012	T	C	CHI-Hu13724_P1, CHI-Hu13724_P2, Case_136, Case_137
**L**	4087	C	T	CHI-Hu13724_P1, CHI-Hu13724_P2, Case_136, Case_137
**L**	4102	T	C	CHI-Hu13724_P1, CHI-Hu13724_P2, Case_136, Case_137
**L**	4105	C	T	CHI-Hu13724_P1, CHI-Hu13724_P2, Case_136, Case_137
**L**	4165	G	A	CHI-Hu13724_P1, CHI-Hu13724_P2, Case_136, Case_137
**L**	4180	G	A	CHI-Hu13724_P1, CHI-Hu13724_P2, Case_136, Case_137
**L**	4216	T	C	CHI-Hu13724_P1, CHI-Hu13724_P2, Case_136, Case_137
**L**	4243	A	G	CHI-Hu13724_P1, CHI-Hu13724_P2, Case_136, Case_137
**L**	4345	T	C	CHI-Hu13724_P1, CHI-Hu13724_P2, Case_136, Case_137
**L**	4384	C	A	CHI-Hu13724_P1, CHI-Hu13724_P2, Case_136, Case_137
**L**	4462	T	C	CHI-Hu13724_P1, CHI-Hu13724_P2, Case_136, Case_137
**L**	4486	G	A	CHI-Hu13724_P1, CHI-Hu13724_P2, Case_136, Case_137
**L**	4507	T	C	CHI-Hu13724_P1, CHI-Hu13724_P2, Case_136, Case_137
**L**	4510	A	T	CHI-Hu13724_P1, CHI-Hu13724_P2, Case_136, Case_137
**L**	4654	A	G	CHI-Hu13724_P1, CHI-Hu13724_P2, Case_136, Case_137
**L**	4666	C	T	CHI-Hu13724_P1, CHI-Hu13724_P2, Case_136, Case_137
**L**	4699	A	G	CHI-Hu13724_P1, CHI-Hu13724_P2, Case_136, Case_137
**L**	4720	G	A	CHI-Hu13724_P1, CHI-Hu13724_P2, Case_136, Case_137
**L**	4721	C	T	CHI-Hu13724_P1, CHI-Hu13724_P2, Case_136, Case_137
**L**	4741	A	G	CHI-Hu13724_P1, CHI-Hu13724_P2, Case_136, Case_137
**L**	4765	G	A	CHI-Hu13724_P1, CHI-Hu13724_P2, Case_136, Case_137
**L**	4777	G	A	CHI-Hu13724_P1, CHI-Hu13724_P2, Case_136, Case_137
**L**	4820	T	C	CHI-Hu13724_P1, CHI-Hu13724_P2, Case_136, Case_137
**L**	4834	T	C	CHI-Hu13724_P1, CHI-Hu13724_P2, Case_136, Case_137
**L**	4888	A	G	CHI-Hu13724_P1, CHI-Hu13724_P2, Case_136, Case_137
**L**	4924	C	T	CHI-Hu13724_P1, CHI-Hu13724_P2, Case_136, Case_137
**L**	4928	C	T	CHI-Hu13724_P1, CHI-Hu13724_P2, Case_136, Case_137

**L**	4975	C	T			CHI-Hu13724_P1, CHI-Hu13724_P2, Case_136, Case_137
**L**	4987	G	A			CHI-Hu13724_P1, CHI-Hu13724_P2, Case_136, Case_137
**L**	4993	C	T			CHI-Hu13724_P1, CHI-Hu13724_P2, Case_136, Case_137
**L**	5008	G	A			CHI-Hu13724_P1, CHI-Hu13724_P2, Case_136, Case_137
**L**	5017	G	A			CHI-Hu13724_P1, CHI-Hu13724_P2, Case_136, Case_137
**L**	5038	C	T			CHI-Hu13724_P1, CHI-Hu13724_P2, Case_136, Case_137
**L**	5056	G	A			CHI-Hu13724_P1, CHI-Hu13724_P2, Case_136, Case_137
**L**	5071	G	A			CHI-Hu13724_P1, CHI-Hu13724_P2, Case_136, Case_137
**L**	5110	T	G			CHI-Hu13724_P1, CHI-Hu13724_P2, Case_136, Case_137
**L**	5143	A	G			CHI-Hu13724_P1, CHI-Hu13724_P2, Case_136, Case_137
**L**	5155	A	G			CHI-Hu13724_P1, CHI-Hu13724_P2, Case_136, Case_137
**L**	5158	T	C			CHI-Hu13724_P1, CHI-Hu13724_P2, Case_136, Case_137
**L**	5188	A	G			CHI-Hu13724_P1, CHI-Hu13724_P2, Case_136, Case_137
**L**	5200	A	G			CHI-Hu13724_P1, CHI-Hu13724_P2, Case_136, Case_137
**L**	5203	A	C			CHI-Hu13724_P1, CHI-Hu13724_P2, Case_136, Case_137
**L**	5212	T	C			CHI-Hu13724_P1, CHI-Hu13724_P2, Case_136, Case_137
**L**	5227	T	C			CHI-Hu13724_P1, CHI-Hu13724_P2, Case_136, Case_137
**L**	5230	C	T			CHI-Hu13724_P1, CHI-Hu13724_P2, Case_136, Case_137
**L**	5236	T	C			CHI-Hu13724_P1, CHI-Hu13724_P2, Case_136, Case_137
**L**	5246	C	T			CHI-Hu13724_P1, CHI-Hu13724_P2, Case_136, Case_137
**L**	5258	C	A			CHI-Hu13724_P1, CHI-Hu13724_P2, Case_136, Case_137
**L**	5260	G	A			CHI-Hu13724_P1, CHI-Hu13724_P2, Case_136, Case_137
**L**	5293	C	T			CHI-Hu13724_P1, CHI-Hu13724_P2, Case_136, Case_137
**L**	5326	G	A			CHI-Hu13724_P1, CHI-Hu13724_P2, Case_136, Case_137
**L**	5344	A	G			CHI-Hu13724_P1, CHI-Hu13724_P2, Case_136, Case_137
**L**	5359	G	A			CHI-Hu13724_P1, CHI-Hu13724_P2, Case_136, Case_137
**L**	5405	C	A			CHI-Hu13724_P1, CHI-Hu13724_P2, Case_136, Case_137
**L**	5518	A	G			CHI-Hu13724_P1, CHI-Hu13724_P2, Case_136, Case_137
**L**	5533	C	T			CHI-Hu13724_P1, CHI-Hu13724_P2, Case_136, Case_137
**L**	5596	A	G			CHI-Hu13724_P1, CHI-Hu13724_P2, Case_136, Case_137
**L**	5614	A	G			CHI-Hu13724_P1, CHI-Hu13724_P2, Case_136, Case_137
**L**	5626	G	A			CHI-Hu13724_P1, CHI-Hu13724_P2, Case_136, Case_137
**L**	5635	C	T			CHI-Hu13724_P1, CHI-Hu13724_P2, Case_136, Case_137
**L**	5638	A	G			CHI-Hu13724_P1, CHI-Hu13724_P2, Case_136, Case_137
**L**	5656	G	A			CHI-Hu13724_P1, CHI-Hu13724_P2, Case_136, Case_137
**L**	5701	C	T			CHI-Hu13724_P1, CHI-Hu13724_P2, Case_136, Case_137
**L**	5710	T	C			CHI-Hu13724_P1, CHI-Hu13724_P2, Case_136, Case_137
**L**	5743	G	A			CHI-Hu13724_P1, CHI-Hu13724_P2, Case_136, Case_137
**L**	5758	T	C			CHI-Hu13724_P1, CHI-Hu13724_P2, Case_136, Case_137
**L**	5803	T	C			CHI-Hu13724_P1, CHI-Hu13724_P2, Case_136, Case_137
**L**	5947	C	T			CHI-Hu13724_P1, CHI-Hu13724_P2, Case_136, Case_137
**L**	5956	C	T			CHI-Hu13724_P1, CHI-Hu13724_P2, Case_136, Case_137
**L**	5968	T	C			CHI-Hu13724_P1, CHI-Hu13724_P2, Case_136, Case_137
**L**	5986	T	C			CHI-Hu13724_P1, CHI-Hu13724_P2, Case_136, Case_137
**L**	5996	C	T			CHI-Hu13724_P1, CHI-Hu13724_P2, Case_136, Case_137
**L**	6028	G	A			CHI-Hu13724_P1, CHI-Hu13724_P2, Case_136, Case_137
**L**	6112	G	A			CHI-Hu13724_P1, CHI-Hu13724_P2, Case_136, Case_137
**L**	6133	A	G			CHI-Hu13724_P1, CHI-Hu13724_P2, Case_136, Case_137
**L**	6142	G	A			CHI-Hu13724_P1, CHI-Hu13724_P2, Case_136, Case_137
**L**	6199	C	T			CHI-Hu13724_P1, CHI-Hu13724_P2, Case_136, Case_137
**L**	6247	T	C			CHI-Hu13724_P1, CHI-Hu13724_P2, Case_136, Case_137
**L**	6250	C	T			CHI-Hu13724_P1, CHI-Hu13724_P2, Case_136, Case_137
**L**	6256	T	C			CHI-Hu13724_P1, CHI-Hu13724_P2, Case_136, Case_137
**L**	6289	T	C			CHI-Hu13724_P1, CHI-Hu13724_P2, Case_136, Case_137
**L**	6298	A	G			CHI-Hu13724_P1, CHI-Hu13724_P2, Case_136, Case_137
**L**	6346	C	T			CHI-Hu13724_P1, CHI-Hu13724_P2, Case_136, Case_137
**L**	6358	C	T			CHI-Hu13724_P1, CHI-Hu13724_P2, Case_136, Case_137
**L**	6373	A	T			CHI-Hu13724_P1, CHI-Hu13724_P2, Case_136, Case_137
**L**	6397	G	A			CHI-Hu13724_P1, CHI-Hu13724_P2, Case_136, Case_137
**L**	6448	C	T			CHI-Hu13724_P1, CHI-Hu13724_P2, Case_136, Case_137
**L**	6449	T	C			CHI-Hu13724_P1, CHI-Hu13724_P2, Case_136, Case_137
**L**	6454	A	G			CHI-Hu13724_P1, CHI-Hu13724_P2, Case_136, Case_137
**L**	6481	A	C			CHI-Hu13724_P1, CHI-Hu13724_P2, Case_136, Case_137

**Compared to Epuyén 2018-19 Patient 1**						
**Segment**	**Position (nt)**	**REF**	**ALT**	**Change N**	**/NSs (+1)**	**Samples with mutation**
**S**	51	C	T			CHI-Hu13724_P1, CHI-Hu13724_P2, Case_136, Case_137
**S**	162	T	C			CHI-Hu13724_P1, CHI-Hu13724_P2, Case_136, Case_137

**S**	169	G	A	S46N	V20I	CHI-Hu13724_P2
**S**	192	T	C			CHI-Hu13724_P1, CHI-Hu13724_P2, Case_136, Case_137
**S**	215	C	T		S35L	CHI-Hu13724_P1, CHI-Hu13724_P2, Case_136, Case_137
**S**	230	G	A		R40Q	CHI-Hu13724_P1, CHI-Hu13724_P2, Case_136, Case_137
**S**	251	G	A		S47N	CHI-Hu13724_P1, CHI-Hu13724_P2, Case_136, Case_137
**S**	344	T	C			CHI-Hu13724_P1, CHI-Hu13724_P2, Case_136, Case_137
**S**	353	C	T			CHI-Hu13724_P1, CHI-Hu13724_P2, Case_136, Case_137
**S**	356	C	T			CHI-Hu13724_P1, CHI-Hu13724_P2, Case_136, Case_137
**S**	357	T	C			CHI-Hu13724_P1, CHI-Hu13724_P2, Case_136, Case_137
**S**	368	G	A			CHI-Hu13724_P1, CHI-Hu13724_P2, Case_136, Case_137
**S**	443	C	T			CHI-Hu13724_P1, CHI-Hu13724_P2, Case_136, Case_137
**S**	455	G	A			CHI-Hu13724_P1, CHI-Hu13724_P2, Case_136, Case_137
**S**	464	C	T			CHI-Hu13724_P1, CHI-Hu13724_P2, Case_136, Case_137
**S**	473	A	G			CHI-Hu13724_P1, CHI-Hu13724_P2, Case_136, Case_137
**S**	488	T	C			CHI-Hu13724_P1, CHI-Hu13724_P2, Case_136, Case_137
**S**	497	T	C			CHI-Hu13724_P1, CHI-Hu13724_P2, Case_136, Case_137
**S**	533	A	G			CHI-Hu13724_P1, CHI-Hu13724_P2, Case_136, Case_137
**S**	551	A	G			CHI-Hu13724_P1, CHI-Hu13724_P2, Case_136, Case_137
**S**	578	G	A			CHI-Hu13724_P1, CHI-Hu13724_P2, Case_136, Case_137
**S**	587	G	A			CHI-Hu13724_P1, CHI-Hu13724_P2, Case_136, Case_137
**S**	623	T	G			CHI-Hu13724_P1, CHI-Hu13724_P2, Case_136, Case_137
**S**	650	C	T			CHI-Hu13724_P1, CHI-Hu13724_P2, Case_136, Case_137
**S**	674	C	T			CHI-Hu13724_P1, CHI-Hu13724_P2, Case_136, Case_137
**S**	683	C	T			CHI-Hu13724_P1, CHI-Hu13724_P2, Case_136, Case_137
**S**	689	A	G			CHI-Hu13724_P1, CHI-Hu13724_P2, Case_136, Case_137
**S**	698	G	A			CHI-Hu13724_P1, CHI-Hu13724_P2, Case_136, Case_137
**S**	719	G	A			CHI-Hu13724_P1, CHI-Hu13724_P2, Case_136, Case_137
**S**	771	T	C			CHI-Hu13724_P1, CHI-Hu13724_P2, Case_136, Case_137
**S**	818	G	A			CHI-Hu13724_P1, CHI-Hu13724_P2, Case_136, Case_137
**S**	839	G	A			CHI-Hu13724_P1, CHI-Hu13724_P2, Case_136, Case_137
**S**	848	C	T			CHI-Hu13724_P1, CHI-Hu13724_P2, Case_136, Case_137
**S**	881	G	A			CHI-Hu13724_P1, CHI-Hu13724_P2, Case_136, Case_137
**S**	962	G	A			CHI-Hu13724_P1, CHI-Hu13724_P2, Case_136, Case_137
**S**	995	C	T			CHI-Hu13724_P1, CHI-Hu13724_P2, Case_136, Case_137
**S**	1064	T	C			CHI-Hu13724_P1, CHI-Hu13724_P2, Case_136, Case_137
**S**	1094	A	G			CHI-Hu13724_P1, CHI-Hu13724_P2, Case_136, Case_137
**S**	1109	C	T			CHI-Hu13724_P1, CHI-Hu13724_P2, Case_136, Case_137
**S**	1127	G	A			CHI-Hu13724_P1, CHI-Hu13724_P2, Case_136, Case_137
**S**	1133	A	G			CHI-Hu13724_P1, CHI-Hu13724_P2, Case_136, Case_137
**S**	1163	A	G			CHI-Hu13724_P1, CHI-Hu13724_P2, Case_136, Case_137
**S**	1166	G	A			CHI-Hu13724_P1, CHI-Hu13724_P2, Case_136, Case_137
**S**	1175	C	T			CHI-Hu13724_P1, CHI-Hu13724_P2, Case_136, Case_137
**S**	1259	A	T			CHI-Hu13724_P1, CHI-Hu13724_P2, Case_136, Case_137
**S**	1271	C	T			CHI-Hu13724_P1, CHI-Hu13724_P2, Case_136, Case_137
**S**	1310	A	T			CHI-Hu13724_P1, CHI-Hu13724_P2, Case_136, Case_137
**S**	1320	A	G			CHI-Hu13724_P1, CHI-Hu13724_P2, Case_136, Case_137
**S**	1322	A	G			CHI-Hu13724_P1, CHI-Hu13724_P2, Case_136, Case_137
**S**	1323	C	T			CHI-Hu13724_P1, CHI-Hu13724_P2, Case_136, Case_137
**S**	1326	A	G			CHI-Hu13724_P1, CHI-Hu13724_P2, Case_136, Case_137
**S**	1334	T	A			CHI-Hu13724_P1, CHI-Hu13724_P2, Case_136, Case_137
**S**	1340	G	T			CHI-Hu13724_P1, CHI-Hu13724_P2, Case_136, Case_137
**S**	1346	G	A			CHI-Hu13724_P1, CHI-Hu13724_P2, Case_136, Case_137
**S**	1387	T	C			CHI-Hu13724_P1, CHI-Hu13724_P2, Case_136, Case_137
**S**	1440	G	A			CHI-Hu13724_P1, CHI-Hu13724_P2, Case_136, Case_137
**S**	1441	A	T			CHI-Hu13724_P1, CHI-Hu13724_P2, Case_136, Case_137
**S**	1472	C	T			CHI-Hu13724_P1, CHI-Hu13724_P2, Case_136, Case_137
**S**	1473	C	T			CHI-Hu13724_P1, CHI-Hu13724_P2, Case_136, Case_137
**S**	1491	A	G			CHI-Hu13724_P1, CHI-Hu13724_P2, Case_136, Case_137
**S**	1492	A	C			CHI-Hu13724_P1, CHI-Hu13724_P2, Case_136, Case_137
**S**	1505	T	C			CHI-Hu13724_P1, CHI-Hu13724_P2, Case_136, Case_137
**S**	1518	C	T			CHI-Hu13724_P1, CHI-Hu13724_P2, Case_136, Case_137
**S**	1548	G	A			CHI-Hu13724_P1, CHI-Hu13724_P2, Case_136, Case_137
**S**	1568	C	T			CHI-Hu13724_P1, CHI-Hu13724_P2, Case_136, Case_137
**S**	1578	T	A			CHI-Hu13724_P1, CHI-Hu13724_P2, Case_136, Case_137
**S**	1599	G	A			CHI-Hu13724_P1, CHI-Hu13724_P2, Case_136, Case_137
**S**	1640	A	G			CHI-Hu13724_P1, CHI-Hu13724_P2, Case_136, Case_137
**S**	1654	G	A			CHI-Hu13724_P1, CHI-Hu13724_P2, Case_136, Case_137
**S**	1656	G	T			CHI-Hu13724_P1, CHI-Hu13724_P2, Case_136, Case_137

**S**	1669	G	A		CHI-Hu13724_P1, CHI-Hu13724_P2, Case_136, Case_137
**S**	1735	C	T		CHI-Hu13724_P1, CHI-Hu13724_P2, Case_136, Case_137
**S**	1790	C	A		CHI-Hu13724_P1, CHI-Hu13724_P2, Case_136, Case_137
**S**	1827	T	C		CHI-Hu13724_P1, CHI-Hu13724_P2, Case_136, Case_137

Segment	Position (nt)	REF	ALT	Change GPC	Samples with mutation
**M**	142	C	T		CHI-Hu13724_P1, CHI-Hu13724_P2, Case_136, Case_137
**M**	154	A	C		CHI-Hu13724_P1, CHI-Hu13724_P2, Case_136, Case_137
**M**	280	C	T		CHI-Hu13724_P1, CHI-Hu13724_P2, Case_136, Case_137
**M**	310	C	T		CHI-Hu13724_P1, CHI-Hu13724_P2, Case_136, Case_137
**M**	319	C	T		CHI-Hu13724_P1, CHI-Hu13724_P2, Case_136, Case_137
**M**	358	C	T		CHI-Hu13724_P1, CHI-Hu13724_P2, Case_136, Case_137
**M**	376	A	G		CHI-Hu13724_P1, CHI-Hu13724_P2, Case_136, Case_137
**M**	386	A	G	I114V	CHI-Hu13724_P1, CHI-Hu13724_P2, Case_136, Case_137
**M**	412	G	A		CHI-Hu13724_P1, CHI-Hu13724_P2, Case_136, Case_137
**M**	427	G	A		CHI-Hu13724_P1, CHI-Hu13724_P2, Case_136, Case_137
**M**	449	T	C		CHI-Hu13724_P1, CHI-Hu13724_P2, Case_136, Case_137
**M**	484	C	T		CHI-Hu13724_P1, CHI-Hu13724_P2, Case_136, Case_137
**M**	508	A	G		CHI-Hu13724_P1, CHI-Hu13724_P2, Case_136, Case_137
**M**	526	T	C		CHI-Hu13724_P1, CHI-Hu13724_P2, Case_136, Case_137
**M**	541	T	G		CHI-Hu13724_P1, CHI-Hu13724_P2, Case_136, Case_137
**M**	559	G	A		CHI-Hu13724_P1, CHI-Hu13724_P2, Case_136, Case_137
**M**	565	C	T		CHI-Hu13724_P1, CHI-Hu13724_P2, Case_136, Case_137
**M**	571	A	G		CHI-Hu13724_P1, CHI-Hu13724_P2, Case_136, Case_137
**M**	628	T	C		CHI-Hu13724_P1, CHI-Hu13724_P2, Case_136, Case_137
**M**	640	A	G		CHI-Hu13724_P1, CHI-Hu13724_P2, Case_136, Case_137
**M**	664	C	T		CHI-Hu13724_P1, CHI-Hu13724_P2, Case_136, Case_137
**M**	670	C	T		CHI-Hu13724_P1, CHI-Hu13724_P2, Case_136, Case_137
**M**	692	C	T	L26F	CHI-Hu13724_P1, CHI-Hu13724_P2, Case_136, Case_137
**M**	694	C	T		CHI-Hu13724_P1, CHI-Hu13724_P2, Case_136, Case_137
**M**	709	G	A		CHI-Hu13724_P1, CHI-Hu13724_P2, Case_136, Case_137
**M**	716	C	T		CHI-Hu13724_P1, CHI-Hu13724_P2, Case_136, Case_137
**M**	718	A	G		CHI-Hu13724_P1, CHI-Hu13724_P2, Case_136, Case_137
**M**	742	A	G		CHI-Hu13724_P1, CHI-Hu13724_P2, Case_136, Case_137
**M**	778	G	A		CHI-Hu13724_P1, CHI-Hu13724_P2, Case_136, Case_137
**M**	793	A	G		CHI-Hu13724_P1, CHI-Hu13724_P2, Case_136, Case_137
**M**	844	C	T		CHI-Hu13724_P1, CHI-Hu13724_P2, Case_136, Case_137
**M**	889	G	A		CHI-Hu13724_P1, CHI-Hu13724_P2, Case_136, Case_137
**M**	895	G	A		CHI-Hu13724_P1, CHI-Hu13724_P2, Case_136, Case_137
**M**	904	C	T		CHI-Hu13724_P1, CHI-Hu13724_P2, Case_136, Case_137
**M**	991	A	G		CHI-Hu13724_P1, CHI-Hu13724_P2, Case_136, Case_137
**M**	1009	C	T		CHI-Hu13724_P1, CHI-Hu13724_P2, Case_136, Case_137
**M**	1033	G	T		CHI-Hu13724_P1, CHI-Hu13724_P2, Case_136, Case_137
**M**	1045	G	A		CHI-Hu13724_P1, CHI-Hu13724_P2, Case_136, Case_137
**M**	1051	A	G		CHI-Hu13724_P1, CHI-Hu13724_P2, Case_136, Case_137
**M**	1078	G	T		CHI-Hu13724_P1, CHI-Hu13724_P2, Case_136, Case_137
**M**	1081	C	A		CHI-Hu13724_P1, CHI-Hu13724_P2, Case_136, Case_137
**M**	1087	G	T		CHI-Hu13724_P1, CHI-Hu13724_P2, Case_136, Case_137
**M**	1096	C	T		CHI-Hu13724_P1, CHI-Hu13724_P2, Case_136, Case_137
**M**	1099	T	C		CHI-Hu13724_P1, CHI-Hu13724_P2, Case_136, Case_137
**M**	1103	A	G	I353V	CHI-Hu13724_P1, CHI-Hu13724_P2, Case_136, Case_137
**M**	1117	A	G		CHI-Hu13724_P1, CHI-Hu13724_P2, Case_136, Case_137
**M**	1159	T	G		CHI-Hu13724_P1, CHI-Hu13724_P2, Case_136, Case_137
**M**	1192	C	T		CHI-Hu13724_P1, CHI-Hu13724_P2, Case_136, Case_137
**M**	1225	A	G		CHI-Hu13724_P1, CHI-Hu13724_P2, Case_136, Case_137
**M**	1237	G	A		CHI-Hu13724_P1, CHI-Hu13724_P2, Case_136, Case_137
**M**	1249	C	T		CHI-Hu13724_P1, CHI-Hu13724_P2, Case_136, Case_137
**M**	1252	C	T		CHI-Hu13724_P1, CHI-Hu13724_P2, Case_136, Case_137
**M**	1255	T	C		CHI-Hu13724_P1, CHI-Hu13724_P2, Case_136, Case_137
**M**	1276	A	C		CHI-Hu13724_P1, CHI-Hu13724_P2, Case_136, Case_137
**M**	1288	G	A		CHI-Hu13724_P1, CHI-Hu13724_P2, Case_136, Case_137
**M**	1300	G	A		CHI-Hu13724_P1, CHI-Hu13724_P2, Case_136, Case_137
**M**	1306	A	G		CHI-Hu13724_P1, CHI-Hu13724_P2, Case_136, Case_137
**M**	1327	C	T		CHI-Hu13724_P1, CHI-Hu13724_P2, Case_136, Case_137
**M**	1393	A	T		CHI-Hu13724_P1, CHI-Hu13724_P2, Case_136, Case_137
**M**	1405	A	G		CHI-Hu13724_P1, CHI-Hu13724_P2, Case_136, Case_137
**M**	1408	A	G		CHI-Hu13724_P1, CHI-Hu13724_P2, Case_136, Case_137
**M**	1465	A	C		CHI-Hu13724_P1, CHI-Hu13724_P2, Case_136, Case_137
**M**	1480	C	T		CHI-Hu13724_P1, CHI-Hu13724_P2, Case_136, Case_137

**M**	1492	G	A		CHI-Hu13724_P1, CHI-Hu13724_P2, Case_136, Case_137
**M**	1498	A	G		CHI-Hu13724_P1, CHI-Hu13724_P2, Case_136, Case_137
**M**	1510	C	A		CHI-Hu13724_P1, CHI-Hu13724_P2, Case_136, Case_137
**M**	1517	T	C		CHI-Hu13724_P1, CHI-Hu13724_P2, Case_136, Case_137
**M**	1525	C	A		CHI-Hu13724_P1, CHI-Hu13724_P2, Case_136, Case_137
**M**	1541	A	G	I499V	CHI-Hu13724_P1, CHI-Hu13724_P2, Case_136, Case_137
**M**	1582	G	A		CHI-Hu13724_P1, CHI-Hu13724_P2, Case_136, Case_137
**M**	1591	G	A		CHI-Hu13724_P1, CHI-Hu13724_P2, Case_136, Case_137
**M**	1705	T	C		CHI-Hu13724_P1, CHI-Hu13724_P2, Case_136, Case_137
**M**	1714	A	G		CHI-Hu13724_P1, CHI-Hu13724_P2, Case_136, Case_137
**M**	1732	A	G		CHI-Hu13724_P1, CHI-Hu13724_P2, Case_136, Case_137
**M**	1771	C	T		CHI-Hu13724_P1, CHI-Hu13724_P2, Case_136, Case_137
**M**	1780	A	G		CHI-Hu13724_P1, CHI-Hu13724_P2, Case_136, Case_137
**M**	1795	C	T		CHI-Hu13724_P1, CHI-Hu13724_P2, Case_136, Case_137
**M**	1798	G	A		CHI-Hu13724_P1, CHI-Hu13724_P2, Case_136, Case_137
**M**	1822	C	T		CHI-Hu13724_P1, CHI-Hu13724_P2, Case_136, Case_137
**M**	1825	C	T		CHI-Hu13724_P1, CHI-Hu13724_P2, Case_136, Case_137
**M**	1840	G	A		CHI-Hu13724_P1, CHI-Hu13724_P2, Case_136, Case_137
**M**	1873	G	A		CHI-Hu13724_P1, CHI-Hu13724_P2, Case_136, Case_137
**M**	1900	C	T		CHI-Hu13724_P1, CHI-Hu13724_P2, Case_136, Case_137
**M**	1909	A	T		CHI-Hu13724_P1, CHI-Hu13724_P2, Case_136, Case_137
**M**	1921	C	A		CHI-Hu13724_P1, CHI-Hu13724_P2, Case_136, Case_137
**M**	1933	A	G		CHI-Hu13724_P1, CHI-Hu13724_P2, Case_136, Case_137
**M**	1963	A	G		CHI-Hu13724_P1, CHI-Hu13724_P2, Case_136, Case_137
**M**	1968	T	C	I641T	CHI-Hu13724_P1, CHI-Hu13724_P2, Case_136, Case_137
**M**	1972	C	T		CHI-Hu13724_P1, CHI-Hu13724_P2, Case_136, Case_137
**M**	1990	T	C		CHI-Hu13724_P1, CHI-Hu13724_P2, Case_136, Case_137
**M**	2009	T	C		CHI-Hu13724_P1, CHI-Hu13724_P2, Case_136, Case_137
**M**	2017	G	A		CHI-Hu13724_P1, CHI-Hu13724_P2, Case_136, Case_137
**M**	2023	T	C		CHI-Hu13724_P1, CHI-Hu13724_P2, Case_136, Case_137
**M**	2089	A	G		CHI-Hu13724_P1, CHI-Hu13724_P2, Case_136, Case_137
**M**	2090	T	C		CHI-Hu13724_P1, CHI-Hu13724_P2, Case_136, Case_137
**M**	2137	A	G		CHI-Hu13724_P1, CHI-Hu13724_P2, Case_136, Case_137
**M**	2143	C	T		CHI-Hu13724_P1, CHI-Hu13724_P2, Case_136, Case_137
**M**	2158	T	C		CHI-Hu13724_P1, CHI-Hu13724_P2, Case_136, Case_137
**M**	2188	A	G		CHI-Hu13724_P1, CHI-Hu13724_P2, Case_136, Case_137
**M**	2236	C	T		CHI-Hu13724_P1, CHI-Hu13724_P2, Case_136, Case_137
**M**	2254	C	T		CHI-Hu13724_P1, CHI-Hu13724_P2, Case_136, Case_137
**M**	2296	G	A		CHI-Hu13724_P1, CHI-Hu13724_P2, Case_136, Case_137
**M**	2404	C	T		CHI-Hu13724_P1, CHI-Hu13724_P2, Case_136, Case_137
**M**	2443	C	A		CHI-Hu13724_P1, CHI-Hu13724_P2, Case_136, Case_137
**M**	2464	A	G		CHI-Hu13724_P1, CHI-Hu13724_P2, Case_136, Case_137
**M**	2518	C	T		CHI-Hu13724_P1, CHI-Hu13724_P2, Case_136, Case_137
**M**	2575	T	C		CHI-Hu13724_P1, CHI-Hu13724_P2, Case_136, Case_137
**M**	2593	T	C		CHI-Hu13724_P1, CHI-Hu13724_P2, Case_136, Case_137
**M**	2596	A	G		CHI-Hu13724_P1, CHI-Hu13724_P2, Case_136, Case_137
**M**	2617	A	G		CHI-Hu13724_P1, CHI-Hu13724_P2, Case_136, Case_137
**M**	2686	C	T		CHI-Hu13724_P1, CHI-Hu13724_P2, Case_136, Case_137
**M**	2692	T	C		CHI-Hu13724_P1, CHI-Hu13724_P2, Case_136, Case_137
**M**	2740	C	T		CHI-Hu13724_P1, CHI-Hu13724_P2, Case_136, Case_137
**M**	2794	T	C		CHI-Hu13724_P1, CHI-Hu13724_P2, Case_136, Case_137
**M**	2858	G	A	A938T	CHI-Hu13724_P1, CHI-Hu13724_P2, Case_136, Case_137
**M**	2893	T	C		CHI-Hu13724_P1, CHI-Hu13724_P2, Case_136, Case_137
**M**	2908	A	C		CHI-Hu13724_P1, CHI-Hu13724_P2, Case_136, Case_137
**M**	2911	T	C		CHI-Hu13724_P1, CHI-Hu13724_P2, Case_136, Case_137
**M**	2959	C	T		CHI-Hu13724_P1, CHI-Hu13724_P2, Case_136, Case_137
**M**	2962	C	T		CHI-Hu13724_P1, CHI-Hu13724_P2, Case_136, Case_137
**M**	2968	A	G		CHI-Hu13724_P1, CHI-Hu13724_P2, Case_136, Case_137
**M**	2974	G	A		CHI-Hu13724_P1, CHI-Hu13724_P2, Case_136, Case_137
**M**	3013	C	A		CHI-Hu13724_P1, CHI-Hu13724_P2, Case_136, Case_137
**M**	3025	T	C		CHI-Hu13724_P1, CHI-Hu13724_P2, Case_136, Case_137
**M**	3037	C	T		CHI-Hu13724_P1, CHI-Hu13724_P2, Case_136, Case_137
**M**	3049	G	A		CHI-Hu13724_P1, CHI-Hu13724_P2, Case_136, Case_137
**M**	3076	A	G		CHI-Hu13724_P1, CHI-Hu13724_P2, Case_136, Case_137
**M**	3085	C	T		CHI-Hu13724_P1, CHI-Hu13724_P2, Case_136, Case_137
**M**	3112	A	G		CHI-Hu13724_P1, CHI-Hu13724_P2, Case_136, Case_137
**M**	3118	C	T		CHI-Hu13724_P1, CHI-Hu13724_P2, Case_136, Case_137
**M**	3127	A	G		CHI-Hu13724_P1, CHI-Hu13724_P2, Case_136, Case_137

**M**	3145	A	G		CHI-Hu13724_P1, CHI-Hu13724_P2, Case_136, Case_137
**M**	3154	T	C		CHI-Hu13724_P1, CHI-Hu13724_P2, Case_136, Case_137
**M**	3205	C	T		CHI-Hu13724_P1, CHI-Hu13724_P2, Case_136, Case_137
**M**	3208	C	T		CHI-Hu13724_P1, CHI-Hu13724_P2, Case_136, Case_137
**M**	3209	T	G	S1055A	CHI-Hu13724_P1, CHI-Hu13724_P2, Case_136, Case_137
**M**	3220	T	A		CHI-Hu13724_P1, CHI-Hu13724_P2, Case_136, Case_137
**M**	3250	A	G		CHI-Hu13724_P1, CHI-Hu13724_P2, Case_136, Case_137
**M**	3310	T	A		CHI-Hu13724_P1, CHI-Hu13724_P2, Case_136, Case_137
**M**	3340	A	G		CHI-Hu13724_P1, CHI-Hu13724_P2, Case_136, Case_137
**M**	3346	T	C		CHI-Hu13724_P1, CHI-Hu13724_P2, Case_136, Case_137
**M**	3367	C	T		CHI-Hu13724_P1, CHI-Hu13724_P2, Case_136, Case_137
**M**	3389	A	G	I1115V	CHI-Hu13724_P1, CHI-Hu13724_P2, Case_136, Case_137
**M**	3409	C	T		CHI-Hu13724_P1, CHI-Hu13724_P2, Case_136, Case_137
**M**	3425	G	A		CHI-Hu13724_P1, CHI-Hu13724_P2, Case_136, Case_137
**M**	3430	C	G		CHI-Hu13724_P1, CHI-Hu13724_P2, Case_136, Case_137
**M**	3460	T	C		CHI-Hu13724_P1, CHI-Hu13724_P2, Case_136, Case_137
**M**	3472	C	T		CHI-Hu13724_P1, CHI-Hu13724_P2, Case_136, Case_137
**M**	3473	T	G		CHI-Hu13724_P1, CHI-Hu13724_P2, Case_136, Case_137
**M**	3477	A	G		CHI-Hu13724_P1, CHI-Hu13724_P2, Case_136, Case_137
**M**	3485	T	A		CHI-Hu13724_P1, CHI-Hu13724_P2, Case_136, Case_137
**M**	3512	A	G		CHI-Hu13724_P1, CHI-Hu13724_P2, Case_136, Case_137
**M**	3518	C	T		CHI-Hu13724_P1, CHI-Hu13724_P2, Case_136, Case_137
**M**	3591	C	T		CHI-Hu13724_P1, CHI-Hu13724_P2, Case_136, Case_137

**Segment**	**Position (nt)**	**REF**	**ALT**	**Change RdRp**	**Samples with mutation**
**L**	48	A	G		CHI-Hu13724_P1, CHI-Hu13724_P2, Case_136, Case_137
**L**	81	G	A		CHI-Hu13724_P1, CHI-Hu13724_P2, Case_136, Case_137
**L**	84	A	G		CHI-Hu13724_P1, CHI-Hu13724_P2, Case_136, Case_137
**L**	132	C	T		CHI-Hu13724_P1, CHI-Hu13724_P2, Case_136, Case_137
**L**	138	C	T		CHI-Hu13724_P1, CHI-Hu13724_P2, Case_136, Case_137
**L**	168	C	T		CHI-Hu13724_P1, CHI-Hu13724_P2, Case_136, Case_137
**L**	210	T	C		CHI-Hu13724_P1, CHI-Hu13724_P2, Case_136, Case_137
**L**	228	A	C		CHI-Hu13724_P1, CHI-Hu13724_P2, Case_136, Case_137
**L**	324	T	C		CHI-Hu13724_P1, CHI-Hu13724_P2, Case_136, Case_137
**L**	333	C	T		CHI-Hu13724_P1, CHI-Hu13724_P2, Case_136, Case_137
**L**	336	C	A		CHI-Hu13724_P1, CHI-Hu13724_P2, Case_136, Case_137
**L**	354	T	C		CHI-Hu13724_P1, CHI-Hu13724_P2, Case_136, Case_137
**L**	369	A	G		CHI-Hu13724_P1, CHI-Hu13724_P2, Case_136, Case_137
**L**	420	T	A		CHI-Hu13724_P1, CHI-Hu13724_P2, Case_136, Case_137
**L**	423	C	T		CHI-Hu13724_P1, CHI-Hu13724_P2, Case_136, Case_137
**L**	429	A	G		CHI-Hu13724_P1, CHI-Hu13724_P2, Case_136, Case_137
**L**	441	C	T		CHI-Hu13724_P1, CHI-Hu13724_P2, Case_136, Case_137
**L**	447	T	C		CHI-Hu13724_P1, CHI-Hu13724_P2, Case_136, Case_137
**L**	450	G	A		CHI-Hu13724_P1, CHI-Hu13724_P2, Case_136, Case_137
**L**	451	G	A	V141I	CHI-Hu13724_P1, CHI-Hu13724_P2, Case_136, Case_137
**L**	461	G	A	R144K	CHI-Hu13724_P1, CHI-Hu13724_P2, Case_136, Case_137
**L**	483	C	T		CHI-Hu13724_P1, CHI-Hu13724_P2, Case_136, Case_137
**L**	504	A	G		CHI-Hu13724_P1, CHI-Hu13724_P2, Case_136, Case_137
**L**	507	G	T		CHI-Hu13724_P1, CHI-Hu13724_P2, Case_136, Case_137
**L**	534	C	T		CHI-Hu13724_P1, CHI-Hu13724_P2, Case_136, Case_137
**L**	555	A	G		CHI-Hu13724_P1, CHI-Hu13724_P2, Case_136, Case_137
**L**	613	A	C		CHI-Hu13724_P1, CHI-Hu13724_P2, Case_136, Case_137
**L**	621	C	T		CHI-Hu13724_P1, CHI-Hu13724_P2, Case_136, Case_137
**L**	649	C	T		CHI-Hu13724_P1, CHI-Hu13724_P2, Case_136, Case_137
**L**	669	A	G		CHI-Hu13724_P1, CHI-Hu13724_P2, Case_136, Case_137
**L**	675	C	T		CHI-Hu13724_P1, CHI-Hu13724_P2, Case_136, Case_137
**L**	708	C	T		CHI-Hu13724_P1, CHI-Hu13724_P2, Case_136, Case_137
**L**	744	A	G		CHI-Hu13724_P1, CHI-Hu13724_P2, Case_136, Case_137
**L**	915	G	A		CHI-Hu13724_P1, CHI-Hu13724_P2, Case_136, Case_137
**L**	921	A	G		CHI-Hu13724_P1, CHI-Hu13724_P2, Case_136, Case_137
**L**	930	G	A		CHI-Hu13724_P1, CHI-Hu13724_P2, Case_136, Case_137
**L**	945	G	A		CHI-Hu13724_P1, CHI-Hu13724_P2, Case_136, Case_137
**L**	954	C	T		CHI-Hu13724_P1, CHI-Hu13724_P2, Case_136, Case_137
**L**	996	C	A		CHI-Hu13724_P1, CHI-Hu13724_P2, Case_136, Case_137
**L**	1011	G	A		CHI-Hu13724_P1, CHI-Hu13724_P2, Case_136, Case_137
**L**	1062	T	C		CHI-Hu13724_P1, CHI-Hu13724_P2, Case_136, Case_137
**L**	1077	A	G		CHI-Hu13724_P1, CHI-Hu13724_P2, Case_136, Case_137
**L**	1083	C	T		CHI-Hu13724_P1, CHI-Hu13724_P2, Case_136, Case_137
**L**	1092	T	C		CHI-Hu13724_P1, CHI-Hu13724_P2, Case_136, Case_137

**L**	1119	C	T		CHI-Hu13724_P1, CHI-Hu13724_P2, Case_136, Case_137
**L**	1121	G	A	S364N	CHI-Hu13724_P1, CHI-Hu13724_P2, Case_136, Case_137
**L**	1134	G	A		CHI-Hu13724_P1, CHI-Hu13724_P2, Case_136, Case_137
**L**	1143	A	G		CHI-Hu13724_P1, CHI-Hu13724_P2, Case_136, Case_137
**L**	1164	A	G		CHI-Hu13724_P1, CHI-Hu13724_P2, Case_136, Case_137
**L**	1173	G	C		CHI-Hu13724_P1, CHI-Hu13724_P2, Case_136, Case_137
**L**	1206	T	C		CHI-Hu13724_P1, CHI-Hu13724_P2, Case_136, Case_137
**L**	1218	G	A		CHI-Hu13724_P1, CHI-Hu13724_P2, Case_136, Case_137
**L**	1221	A	G		CHI-Hu13724_P1, CHI-Hu13724_P2, Case_136, Case_137
**L**	1234	A	G	I402V	CHI-Hu13724_P1, CHI-Hu13724_P2, Case_136, Case_137
**L**	1236	T	C		CHI-Hu13724_P1, CHI-Hu13724_P2, Case_136, Case_137
**L**	1245	T	C		CHI-Hu13724_P1, CHI-Hu13724_P2, Case_136, Case_137
**L**	1254	A	G		CHI-Hu13724_P1, CHI-Hu13724_P2, Case_136, Case_137
**L**	1269	G	A		CHI-Hu13724_P1, CHI-Hu13724_P2, Case_136, Case_137
**L**	1302	C	T		CHI-Hu13724_P1, CHI-Hu13724_P2, Case_136, Case_137
**L**	1305	A	G		CHI-Hu13724_P1, CHI-Hu13724_P2, Case_136, Case_137
**L**	1308	T	A		CHI-Hu13724_P1, CHI-Hu13724_P2, Case_136, Case_137
**L**	1323	C	T		CHI-Hu13724_P1, CHI-Hu13724_P2, Case_136, Case_137
**L**	1371	C	T		CHI-Hu13724_P1, CHI-Hu13724_P2, Case_136, Case_137
**L**	1374	A	G		CHI-Hu13724_P1, CHI-Hu13724_P2, Case_136, Case_137
**L**	1377	T	C		CHI-Hu13724_P1, CHI-Hu13724_P2, Case_136, Case_137
**L**	1431	T	C		CHI-Hu13724_P1, CHI-Hu13724_P2, Case_136, Case_137
**L**	1465	T	C		CHI-Hu13724_P1, CHI-Hu13724_P2, Case_136, Case_137
**L**	1533	G	A		CHI-Hu13724_P1, CHI-Hu13724_P2, Case_136, Case_137
**L**	1561	T	C		CHI-Hu13724_P1, CHI-Hu13724_P2, Case_136, Case_137
**L**	1587	G	A		CHI-Hu13724_P1, CHI-Hu13724_P2, Case_136, Case_137
**L**	1611	G	A		CHI-Hu13724_P1, CHI-Hu13724_P2, Case_136, Case_137
**L**	1617	C	T		CHI-Hu13724_P1, CHI-Hu13724_P2, Case_136, Case_137
**L**	1626	C	T		CHI-Hu13724_P1, CHI-Hu13724_P2, Case_136, Case_137
**L**	1632	C	T		CHI-Hu13724_P1, CHI-Hu13724_P2, Case_136, Case_137
**L**	1644	G	A		CHI-Hu13724_P1, CHI-Hu13724_P2, Case_136, Case_137
**L**	1647	T	A		CHI-Hu13724_P1, CHI-Hu13724_P2, Case_136, Case_137
**L**	1652	C	T	A541V	CHI-Hu13724_P1, CHI-Hu13724_P2, Case_136, Case_137
**L**	1656	C	T		CHI-Hu13724_P1, CHI-Hu13724_P2, Case_136, Case_137
**L**	1671	A	G		CHI-Hu13724_P1, CHI-Hu13724_P2, Case_136, Case_137
**L**	1674	T	C		CHI-Hu13724_P1, CHI-Hu13724_P2, Case_136, Case_137
**L**	1701	T	C		CHI-Hu13724_P1, CHI-Hu13724_P2, Case_136, Case_137
**L**	1704	T	C		CHI-Hu13724_P1, CHI-Hu13724_P2, Case_136, Case_137
**L**	1719	C	T		CHI-Hu13724_P1, CHI-Hu13724_P2, Case_136, Case_137
**L**	1731	G	A		CHI-Hu13724_P1, CHI-Hu13724_P2, Case_136, Case_137
**L**	1732	C	T		CHI-Hu13724_P1, CHI-Hu13724_P2, Case_136, Case_137
**L**	1734	A	G		CHI-Hu13724_P1, CHI-Hu13724_P2, Case_136, Case_137
**L**	1737	A	G		CHI-Hu13724_P1, CHI-Hu13724_P2, Case_136, Case_137
**L**	1743	G	A		CHI-Hu13724_P1, CHI-Hu13724_P2, Case_136, Case_137
**L**	1761	G	A		CHI-Hu13724_P1, CHI-Hu13724_P2, Case_136, Case_137
**L**	1771	G	T	A581S	CHI-Hu13724_P1, CHI-Hu13724_P2, Case_136, Case_137
**L**	1776	A	G		CHI-Hu13724_P1, CHI-Hu13724_P2, Case_136, Case_137
**L**	1788	C	T		CHI-Hu13724_P1, CHI-Hu13724_P2, Case_136, Case_137
**L**	1812	C	T		CHI-Hu13724_P1, CHI-Hu13724_P2, Case_136, Case_137
**L**	1836	A	G		CHI-Hu13724_P1, CHI-Hu13724_P2, Case_136, Case_137
**L**	1893	C	T		CHI-Hu13724_P1, CHI-Hu13724_P2, Case_136, Case_137
**L**	1902	T	C		CHI-Hu13724_P1, CHI-Hu13724_P2, Case_136, Case_137
**L**	1956	A	G		CHI-Hu13724_P1, CHI-Hu13724_P2, Case_136, Case_137
**L**	1959	G	A		CHI-Hu13724_P1, CHI-Hu13724_P2, Case_136, Case_137
**L**	1960	C	T		CHI-Hu13724_P1, CHI-Hu13724_P2, Case_136, Case_137
**L**	1968	A	G		CHI-Hu13724_P1, CHI-Hu13724_P2, Case_136, Case_137
**L**	1986	A	T		CHI-Hu13724_P1, CHI-Hu13724_P2, Case_136, Case_137
**L**	2115	G	T		CHI-Hu13724_P1, CHI-Hu13724_P2, Case_136, Case_137
**L**	2118	T	C		CHI-Hu13724_P1, CHI-Hu13724_P2, Case_136, Case_137
**L**	2160	C	T		CHI-Hu13724_P1, CHI-Hu13724_P2, Case_136, Case_137
**L**	2169	C	T		CHI-Hu13724_P1, CHI-Hu13724_P2, Case_136, Case_137
**L**	2172	C	T		CHI-Hu13724_P1, CHI-Hu13724_P2, Case_136, Case_137
**L**	2178	T	C		CHI-Hu13724_P1, CHI-Hu13724_P2, Case_136, Case_137
**L**	2179	C	T		CHI-Hu13724_P1, CHI-Hu13724_P2, Case_136, Case_137
**L**	2205	T	C		CHI-Hu13724_P1, CHI-Hu13724_P2, Case_136, Case_137
**L**	2236	C	T		CHI-Hu13724_P1, CHI-Hu13724_P2, Case_136, Case_137
**L**	2238	A	G		CHI-Hu13724_P1, CHI-Hu13724_P2, Case_136, Case_137
**L**	2241	A	G		CHI-Hu13724_P1, CHI-Hu13724_P2, Case_136, Case_137

**L**	2250	C	T		CHI-Hu13724_P1, CHI-Hu13724_P2, Case_136, Case_137
**L**	2253	A	G		CHI-Hu13724_P1, CHI-Hu13724_P2, Case_136, Case_137
**L**	2271	T	C		CHI-Hu13724_P1, CHI-Hu13724_P2, Case_136, Case_137
**L**	2310	C	T		CHI-Hu13724_P1, CHI-Hu13724_P2, Case_136, Case_137
**L**	2323	C	T		CHI-Hu13724_P1, CHI-Hu13724_P2, Case_136, Case_137
**L**	2331	A	G		CHI-Hu13724_P1, CHI-Hu13724_P2, Case_136, Case_137
**L**	2340	A	G		CHI-Hu13724_P1, CHI-Hu13724_P2, Case_136, Case_137
**L**	2355	C	T		CHI-Hu13724_P1, CHI-Hu13724_P2, Case_136, Case_137
**L**	2358	T	C		CHI-Hu13724_P1, CHI-Hu13724_P2, Case_136, Case_137
**L**	2385	C	T		CHI-Hu13724_P1, CHI-Hu13724_P2, Case_136, Case_137
**L**	2397	G	A		CHI-Hu13724_P1, CHI-Hu13724_P2, Case_136, Case_137
**L**	2400	T	G		CHI-Hu13724_P1, CHI-Hu13724_P2, Case_136, Case_137
**L**	2403	A	G		CHI-Hu13724_P1, CHI-Hu13724_P2, Case_136, Case_137
**L**	2418	A	G		CHI-Hu13724_P1, CHI-Hu13724_P2, Case_136, Case_137
**L**	2463	C	T		CHI-Hu13724_P1, CHI-Hu13724_P2, Case_136, Case_137
**L**	2496	C	T		CHI-Hu13724_P1, CHI-Hu13724_P2, Case_136, Case_137
**L**	2520	C	T		CHI-Hu13724_P1, CHI-Hu13724_P2, Case_136, Case_137
**L**	2574	A	G		CHI-Hu13724_P1, CHI-Hu13724_P2, Case_136, Case_137
**L**	2607	A	G		CHI-Hu13724_P1, CHI-Hu13724_P2, Case_136, Case_137
**L**	2616	T	C		CHI-Hu13724_P1, CHI-Hu13724_P2, Case_136, Case_137
**L**	2656	G	T	A876S	CHI-Hu13724_P1, CHI-Hu13724_P2, Case_136, Case_137
**L**	2685	A	G		CHI-Hu13724_P1, CHI-Hu13724_P2, Case_136, Case_137
**L**	2715	T	C		CHI-Hu13724_P1, CHI-Hu13724_P2, Case_136, Case_137
**L**	2721	T	C		CHI-Hu13724_P1, CHI-Hu13724_P2, Case_136, Case_137
**L**	2748	G	A		CHI-Hu13724_P1, CHI-Hu13724_P2, Case_136, Case_137
**L**	2769	C	T		CHI-Hu13724_P1, CHI-Hu13724_P2, Case_136, Case_137
**L**	2775	A	G		CHI-Hu13724_P1, CHI-Hu13724_P2, Case_136, Case_137
**L**	2778	T	C		CHI-Hu13724_P1, CHI-Hu13724_P2, Case_136, Case_137
**L**	2841	G	A		CHI-Hu13724_P1, CHI-Hu13724_P2, Case_136, Case_137
**L**	2853	G	A		CHI-Hu13724_P1, CHI-Hu13724_P2, Case_136, Case_137
**L**	2877	G	A		CHI-Hu13724_P1, CHI-Hu13724_P2, Case_136, Case_137
**L**	2913	C	T		CHI-Hu13724_P1, CHI-Hu13724_P2, Case_136, Case_137
**L**	2982	C	T		CHI-Hu13724_P1, CHI-Hu13724_P2, Case_136, Case_137
**L**	2988	C	T		CHI-Hu13724_P1, CHI-Hu13724_P2, Case_136, Case_137
**L**	3027	C	T		CHI-Hu13724_P1, CHI-Hu13724_P2, Case_136, Case_137
**L**	3036	A	G		CHI-Hu13724_P1, CHI-Hu13724_P2, Case_136, Case_137
**L**	3060	A	G		CHI-Hu13724_P1, CHI-Hu13724_P2, Case_136, Case_137
**L**	3066	T	C		CHI-Hu13724_P1, CHI-Hu13724_P2, Case_136, Case_137
**L**	3096	A	G		CHI-Hu13724_P1, CHI-Hu13724_P2, Case_136, Case_137
**L**	3159	G	A		CHI-Hu13724_P1, CHI-Hu13724_P2, Case_136, Case_137
**L**	3183	C	T		CHI-Hu13724_P1, CHI-Hu13724_P2, Case_136, Case_137
**L**	3186	T	C		CHI-Hu13724_P1, CHI-Hu13724_P2, Case_136, Case_137
**L**	3189	G	A		CHI-Hu13724_P1, CHI-Hu13724_P2, Case_136, Case_137
**L**	3210	G	A		CHI-Hu13724_P1, CHI-Hu13724_P2, Case_136, Case_137
**L**	3231	G	A		CHI-Hu13724_P1, CHI-Hu13724_P2, Case_136, Case_137
**L**	3267	G	A		CHI-Hu13724_P1, CHI-Hu13724_P2, Case_136, Case_137
**L**	3297	G	A		CHI-Hu13724_P1, CHI-Hu13724_P2, Case_136, Case_137
**L**	3300	C	T		CHI-Hu13724_P1, CHI-Hu13724_P2, Case_136, Case_137
**L**	3352	C	T		CHI-Hu13724_P1, CHI-Hu13724_P2, Case_136, Case_137
**L**	3393	A	G		CHI-Hu13724_P1, CHI-Hu13724_P2, Case_136, Case_137
**L**	3408	A	G		CHI-Hu13724_P1, CHI-Hu13724_P2, Case_136, Case_137
**L**	3417	C	T		CHI-Hu13724_P1, CHI-Hu13724_P2, Case_136, Case_137
**L**	3483	A	G		CHI-Hu13724_P1, CHI-Hu13724_P2, Case_136, Case_137
**L**	3492	C	T		CHI-Hu13724_P1, CHI-Hu13724_P2, Case_136, Case_137
**L**	3525	G	T		CHI-Hu13724_P1, CHI-Hu13724_P2, Case_136, Case_137
**L**	3531	C	T		CHI-Hu13724_P1, CHI-Hu13724_P2, Case_136, Case_137
**L**	3555	T	C		CHI-Hu13724_P1, CHI-Hu13724_P2, Case_136, Case_137
**L**	3558	C	T		CHI-Hu13724_P1, CHI-Hu13724_P2, Case_136, Case_137
**L**	3564	A	G		CHI-Hu13724_P1, CHI-Hu13724_P2, Case_136, Case_137
**L**	3582	T	C		CHI-Hu13724_P1, CHI-Hu13724_P2, Case_136, Case_137
**L**	3585	C	T		CHI-Hu13724_P1, CHI-Hu13724_P2, Case_136, Case_137
**L**	3606	C	T		CHI-Hu13724_P1, CHI-Hu13724_P2, Case_136, Case_137
**L**	3633	T	C		CHI-Hu13724_P1, CHI-Hu13724_P2, Case_136, Case_137
**L**	3648	G	A		CHI-Hu13724_P1, CHI-Hu13724_P2, Case_136, Case_137
**L**	3663	C	T		CHI-Hu13724_P1, CHI-Hu13724_P2, Case_136, Case_137
**L**	3681	G	A		CHI-Hu13724_P1, CHI-Hu13724_P2, Case_136, Case_137
**L**	3687	A	G		CHI-Hu13724_P1, CHI-Hu13724_P2, Case_136, Case_137
**L**	3702	G	A		CHI-Hu13724_P1, CHI-Hu13724_P2, Case_136, Case_137

**L**	3717	T	A		CHI-Hu13724_P1, CHI-Hu13724_P2, Case_136, Case_137
**L**	3774	G	A		CHI-Hu13724_P1, CHI-Hu13724_P2, Case_136, Case_137
**L**	3843	G	A		CHI-Hu13724_P1, CHI-Hu13724_P2, Case_136, Case_137
**L**	3891	G	A		CHI-Hu13724_P1, CHI-Hu13724_P2, Case_136, Case_137
**L**	3898	T	C		CHI-Hu13724_P1, CHI-Hu13724_P2, Case_136, Case_137
**L**	3954	C	A		CHI-Hu13724_P1, CHI-Hu13724_P2, Case_136, Case_137
**L**	3960	T	C		CHI-Hu13724_P1, CHI-Hu13724_P2, Case_136, Case_137
**L**	4005	T	C		CHI-Hu13724_P1, CHI-Hu13724_P2, Case_136, Case_137
**L**	4050	G	A		CHI-Hu13724_P1, CHI-Hu13724_P2, Case_136, Case_137
**L**	4098	T	C		CHI-Hu13724_P1, CHI-Hu13724_P2, Case_136, Case_137
**L**	4113	A	G		CHI-Hu13724_P1, CHI-Hu13724_P2, Case_136, Case_137
**L**	4161	G	A		CHI-Hu13724_P1, CHI-Hu13724_P2, Case_136, Case_137
**L**	4167	C	T		CHI-Hu13724_P1, CHI-Hu13724_P2, Case_136, Case_137
**L**	4341	T	C		CHI-Hu13724_P1, CHI-Hu13724_P2, Case_136, Case_137
**L**	4349	A	G	N1440S	CHI-Hu13724_P1, CHI-Hu13724_P2, Case_136, Case_137
**L**	4380	C	A		CHI-Hu13724_P1, CHI-Hu13724_P2, Case_136, Case_137
**L**	4431	T	C		CHI-Hu13724_P1, CHI-Hu13724_P2, Case_136, Case_137
**L**	4470	G	A		CHI-Hu13724_P1, CHI-Hu13724_P2, Case_136, Case_137
**L**	4482	G	A		CHI-Hu13724_P1, CHI-Hu13724_P2, Case_136, Case_137
**L**	4545	C	T		CHI-Hu13724_P1, CHI-Hu13724_P2, Case_136, Case_137
**L**	4551	A	G		CHI-Hu13724_P1, CHI-Hu13724_P2, Case_136, Case_137
**L**	4557	T	C		CHI-Hu13724_P1, CHI-Hu13724_P2, Case_136, Case_137
**L**	4572	T	C		CHI-Hu13724_P1, CHI-Hu13724_P2, Case_136, Case_137
**L**	4617	G	A		CHI-Hu13724_P1, CHI-Hu13724_P2, Case_136, Case_137
**L**	4620	G	A		CHI-Hu13724_P1, CHI-Hu13724_P2, Case_136, Case_137
**L**	4656	A	G		CHI-Hu13724_P1, CHI-Hu13724_P2, Case_136, Case_137
**L**	4662	C	T		CHI-Hu13724_P1, CHI-Hu13724_P2, Case_136, Case_137
**L**	4665	A	G		CHI-Hu13724_P1, CHI-Hu13724_P2, Case_136, Case_137
**L**	4674	A	G		CHI-Hu13724_P1, CHI-Hu13724_P2, Case_136, Case_137
**L**	4680	C	T		CHI-Hu13724_P1, CHI-Hu13724_P2, Case_136, Case_137
**L**	4707	T	C		CHI-Hu13724_P1, CHI-Hu13724_P2, Case_136, Case_137
**L**	4716	G	A		CHI-Hu13724_P1, CHI-Hu13724_P2, Case_136, Case_137
**L**	4717	C	T		CHI-Hu13724_P1, CHI-Hu13724_P2, Case_136, Case_137
**L**	4755	T	G		CHI-Hu13724_P1, CHI-Hu13724_P2, Case_136, Case_137
**L**	4773	G	A		CHI-Hu13724_P1, CHI-Hu13724_P2, Case_136, Case_137
**L**	4818	A	G		CHI-Hu13724_P1, CHI-Hu13724_P2, Case_136, Case_137
**L**	4830	T	C		CHI-Hu13724_P1, CHI-Hu13724_P2, Case_136, Case_137
**L**	4855	T	C		CHI-Hu13724_P1, CHI-Hu13724_P2, Case_136, Case_137
**L**	4875	C	T		CHI-Hu13724_P1, CHI-Hu13724_P2, Case_136, Case_137
**L**	4899	A	G		CHI-Hu13724_P1, CHI-Hu13724_P2, Case_136, Case_137
**L**	4905	T	C		CHI-Hu13724_P1, CHI-Hu13724_P2, Case_136, Case_137
**L**	4924	C	T		CHI-Hu13724_P1, CHI-Hu13724_P2, Case_136, Case_137
**L**	4971	C	T		CHI-Hu13724_P1, CHI-Hu13724_P2, Case_136, Case_137
**L**	4974	G	A		CHI-Hu13724_P1, CHI-Hu13724_P2, Case_136, Case_137
**L**	4992	A	G		CHI-Hu13724_P1, CHI-Hu13724_P2, Case_136, Case_137
**L**	4995	C	T		CHI-Hu13724_P1, CHI-Hu13724_P2, Case_136, Case_137
**L**	5013	G	A		CHI-Hu13724_P1, CHI-Hu13724_P2, Case_136, Case_137
**L**	5023	G	A	V1665I	CHI-Hu13724_P1, CHI-Hu13724_P2, Case_136, Case_137
**L**	5052	G	A		CHI-Hu13724_P1, CHI-Hu13724_P2, Case_136, Case_137
**L**	5073	G	A		CHI-Hu13724_P1, CHI-Hu13724_P2, Case_136, Case_137
**L**	5100	A	G		CHI-Hu13724_P1, CHI-Hu13724_P2, Case_136, Case_137
**L**	5106	T	G		CHI-Hu13724_P1, CHI-Hu13724_P2, Case_136, Case_137
**L**	5118	T	G		CHI-Hu13724_P1, CHI-Hu13724_P2, Case_136, Case_137
**L**	5139	A	G		CHI-Hu13724_P1, CHI-Hu13724_P2, Case_136, Case_137
**L**	5151	A	G		CHI-Hu13724_P1, CHI-Hu13724_P2, Case_136, Case_137
**L**	5154	T	C		CHI-Hu13724_P1, CHI-Hu13724_P2, Case_136, Case_137
**L**	5181	G	A		CHI-Hu13724_P1, CHI-Hu13724_P2, Case_136, Case_137
**L**	5196	A	G		CHI-Hu13724_P1, CHI-Hu13724_P2, Case_136, Case_137
**L**	5199	A	C		CHI-Hu13724_P1, CHI-Hu13724_P2, Case_136, Case_137
**L**	5208	T	C		CHI-Hu13724_P1, CHI-Hu13724_P2, Case_136, Case_137
**L**	5223	T	C		CHI-Hu13724_P1, CHI-Hu13724_P2, Case_136, Case_137
**L**	5241	T	C		CHI-Hu13724_P1, CHI-Hu13724_P2, Case_136, Case_137
**L**	5242	C	T		CHI-Hu13724_P1, CHI-Hu13724_P2, Case_136, Case_137
**L**	5247	T	C		CHI-Hu13724_P1, CHI-Hu13724_P2, Case_136, Case_137
**L**	5256	G	A		CHI-Hu13724_P1, CHI-Hu13724_P2, Case_136, Case_137
**L**	5265	T	C		CHI-Hu13724_P1, CHI-Hu13724_P2, Case_136, Case_137
**L**	5283	A	G		CHI-Hu13724_P1, CHI-Hu13724_P2, Case_136, Case_137
**L**	5289	C	T		CHI-Hu13724_P1, CHI-Hu13724_P2, Case_136, Case_137

**L**	5301	G	T		CHI-Hu13724_P1, CHI-Hu13724_P2, Case_136, Case_137
**L**	5304	C	T		CHI-Hu13724_P1, CHI-Hu13724_P2, Case_136, Case_137
**L**	5310	A	G		CHI-Hu13724_P1, CHI-Hu13724_P2, Case_136, Case_137
**L**	5322	G	A		CHI-Hu13724_P1, CHI-Hu13724_P2, Case_136, Case_137
**L**	5340	A	G		CHI-Hu13724_P1, CHI-Hu13724_P2, Case_136, Case_137
**L**	5355	G	A		CHI-Hu13724_P1, CHI-Hu13724_P2, Case_136, Case_137
**L**	5388	G	A		CHI-Hu13724_P1, CHI-Hu13724_P2, Case_136, Case_137
**L**	5401	C	A		CHI-Hu13724_P1, CHI-Hu13724_P2, Case_136, Case_137
**L**	5418	C	T		CHI-Hu13724_P1, CHI-Hu13724_P2, Case_136, Case_137
**L**	5445	C	T		CHI-Hu13724_P1, CHI-Hu13724_P2, Case_136, Case_137
**L**	5457	A	G		CHI-Hu13724_P1, CHI-Hu13724_P2, Case_136, Case_137
**L**	5499	C	T		CHI-Hu13724_P1, CHI-Hu13724_P2, Case_136, Case_137
**L**	5514	A	G		CHI-Hu13724_P1, CHI-Hu13724_P2, Case_136, Case_137
**L**	5520	C	T		CHI-Hu13724_P1, CHI-Hu13724_P2, Case_136, Case_137
**L**	5529	C	T		CHI-Hu13724_P1, CHI-Hu13724_P2, Case_136, Case_137
**L**	5550	C	T		CHI-Hu13724_P1, CHI-Hu13724_P2, Case_136, Case_137
**L**	5610	A	G		CHI-Hu13724_P1, CHI-Hu13724_P2, Case_136, Case_137
**L**	5622	G	A		CHI-Hu13724_P1, CHI-Hu13724_P2, Case_136, Case_137
**L**	5640	G	A		CHI-Hu13724_P1, CHI-Hu13724_P2, Case_136, Case_137
**L**	5652	G	A		CHI-Hu13724_P1, CHI-Hu13724_P2, Case_136, Case_137
**L**	5694	C	T		CHI-Hu13724_P1, CHI-Hu13724_P2, Case_136, Case_137
**L**	5697	C	T		CHI-Hu13724_P1, CHI-Hu13724_P2, Case_136, Case_137
**L**	5721	T	C		CHI-Hu13724_P1, CHI-Hu13724_P2, Case_136, Case_137
**L**	5730	C	T		CHI-Hu13724_P1, CHI-Hu13724_P2, Case_136, Case_137
**L**	5787	C	T		CHI-Hu13724_P1, CHI-Hu13724_P2, Case_136, Case_137
**L**	5799	T	C		CHI-Hu13724_P1, CHI-Hu13724_P2, Case_136, Case_137
**L**	5925	C	G	H1965Q	CHI-Hu13724_P1, CHI-Hu13724_P2, Case_136, Case_137
**L**	5964	A	C		CHI-Hu13724_P1, CHI-Hu13724_P2, Case_136, Case_137
**L**	5991	G	A		CHI-Hu13724_P1, CHI-Hu13724_P2, Case_136, Case_137
**L**	5992	C	T		CHI-Hu13724_P1, CHI-Hu13724_P2, Case_136, Case_137
**L**	6024	G	A		CHI-Hu13724_P1, CHI-Hu13724_P2, Case_136, Case_137
**L**	6030	A	G		CHI-Hu13724_P1, CHI-Hu13724_P2, Case_136, Case_137
**L**	6045	C	T		CHI-Hu13724_P1, CHI-Hu13724_P2, Case_136, Case_137
**L**	6105	C	T		CHI-Hu13724_P1, CHI-Hu13724_P2, Case_136, Case_137
**L**	6108	G	A		CHI-Hu13724_P1, CHI-Hu13724_P2, Case_136, Case_137
**L**	6117	G	T		CHI-Hu13724_P1, CHI-Hu13724_P2, Case_136, Case_137
**L**	6120	A	G		CHI-Hu13724_P1, CHI-Hu13724_P2, Case_136, Case_137
**L**	6129	A	G		CHI-Hu13724_P1, CHI-Hu13724_P2, Case_136, Case_137
**L**	6138	G	A		CHI-Hu13724_P1, CHI-Hu13724_P2, Case_136, Case_137
**L**	6186	T	C		CHI-Hu13724_P1, CHI-Hu13724_P2, Case_136, Case_137
**L**	6195	C	T		CHI-Hu13724_P1, CHI-Hu13724_P2, Case_136, Case_137
**L**	6201	T	C		CHI-Hu13724_P1, CHI-Hu13724_P2, Case_136, Case_137
**L**	6214	T	C		CHI-Hu13724_P1, CHI-Hu13724_P2, Case_136, Case_137
**L**	6252	T	C		CHI-Hu13724_P1, CHI-Hu13724_P2, Case_136, Case_137
**L**	6271	T	C		CHI-Hu13724_P1, CHI-Hu13724_P2, Case_136, Case_137
**L**	6276	T	C		CHI-Hu13724_P1, CHI-Hu13724_P2, Case_136, Case_137
**L**	6285	T	C		CHI-Hu13724_P1, CHI-Hu13724_P2, Case_136, Case_137
**L**	6294	A	G		CHI-Hu13724_P1, CHI-Hu13724_P2, Case_136, Case_137
**L**	6312	A	G		CHI-Hu13724_P1, CHI-Hu13724_P2, Case_136, Case_137
**L**	6330	G	A		CHI-Hu13724_P1, CHI-Hu13724_P2, Case_136, Case_137
**L**	6342	C	T		CHI-Hu13724_P1, CHI-Hu13724_P2, Case_136, Case_137
**L**	6367	A	G	T2113A	CHI-Hu13724_P1, CHI-Hu13724_P2, Case_136, Case_137
**L**	6369	A	T		CHI-Hu13724_P1, CHI-Hu13724_P2, Case_136, Case_137
**L**	6429	T	C		CHI-Hu13724_P1, CHI-Hu13724_P2, Case_136, Case_137
**L**	6445	T	C		CHI-Hu13724_P1, CHI-Hu13724_P2, Case_136, Case_137
**L**	6450	A	G		CHI-Hu13724_P1, CHI-Hu13724_P2, Case_136, Case_137
**L**	6462	T	C		CHI-Hu13724_P1, CHI-Hu13724_P2, Case_136, Case_137
**L**	6477	A	C		CHI-Hu13724_P1, CHI-Hu13724_P2, Case_136, Case_137
