# Supplementary material for: New Andes virus isolate haplotype obtained during prospective close contacts follow-up of an Hantavirus cardiopulmonary syndrome fatal case, Chile
Source: Curr Res Microb Sci. 2025 Sep 16;9:100472. doi: 10.1016/j.crmicr.2025.100472 (PMC12506574; doi:10.1016/j.crmicr.2025.100472)
Supplement: Supplementary file 1 [file mmc1.zip › CRMS_SuppTable3.pdf]

Case	Days post symptoms onset	IgM (Mean ratio ± SD)	IgG (Mean UR/mL ± SD)
136	4	3,494 ± 0,041	129,0 ± 6,221
	8	3,833 ± 0,195	320,6 ± 3,285
	20	2,212 ± 0,089	367,1 ± 1,442
	60	0,716 ± 0,002	436,6 ± 5,367
137	1	0,331 ± 0,001	4,702 ± 0,689
	4	4,683 ± 0,028	342,4 ± 16,18
	6	4,736 ± 0,158	339,6 ± 20,11
	20	4,159 ± 0,067	363,1 ± 13,06
	60	0,931 ± 0,021	412,7 ± 4,513
