# Supplementary material for: New Andes virus isolate haplotype obtained during prospective close contacts follow-up of an Hantavirus cardiopulmonary syndrome fatal case, Chile
Source: Curr Res Microb Sci. 2025 Sep 16;9:100472. doi: 10.1016/j.crmicr.2025.100472 (PMC12506574; doi:10.1016/j.crmicr.2025.100472)

**Supporting Information**

**Supplementary Table 1. Average sequencing depth and genome coverage of Andes virus genomic segments across samples.**

**Supplementary Table 2. Complete list of nucleotide and amino acid substitutions of CHI-Hu13724 compared to reference ANDV genomes CHI-7913 and Epuyén 2018-19 Patient 1.**

**Supplementary Figure 1. Phylogenetic analysis of the M and L segments of CHI-Hu13724 ANDV and other Hantaviruses.** Maximum likelihood phylogeny of full-length M (A) and L (B) segment sequences (~3654 and 6549 bp, respectively) from multiple Andes viruses and other hantavirus. Sequences corresponding to the Chilean isolate CHI-Hu13724 (passages P1 and P2), case 137 and case 136 are highlighted in light purple.


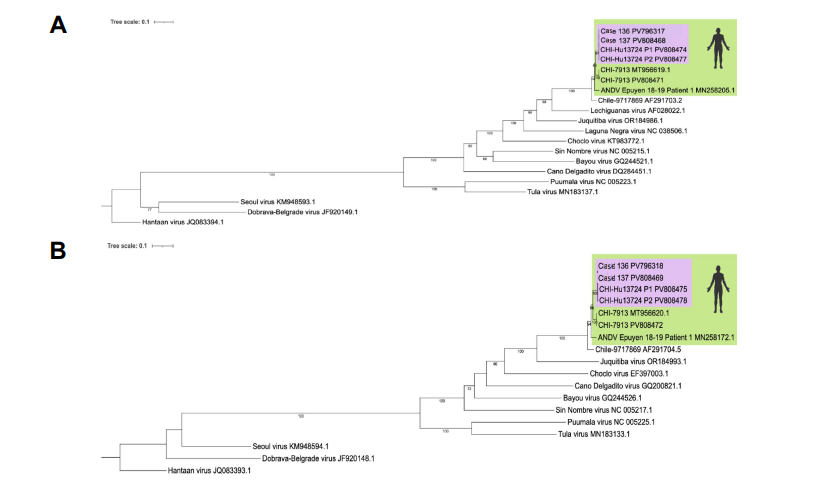

Supplement: Supplementary file 1 [file mmc1.zip › Supporting Information.docx]
