# Supplementary figures and images for: New Andes virus isolate haplotype obtained during prospective close contacts follow-up of an Hantavirus cardiopulmonary syndrome fatal case, Chile
Source: Curr Res Microb Sci. 2025 Sep 16;9:100472. doi: 10.1016/j.crmicr.2025.100472 (PMC12506574; doi:10.1016/j.crmicr.2025.100472)

# Supplementary Figure 1

A

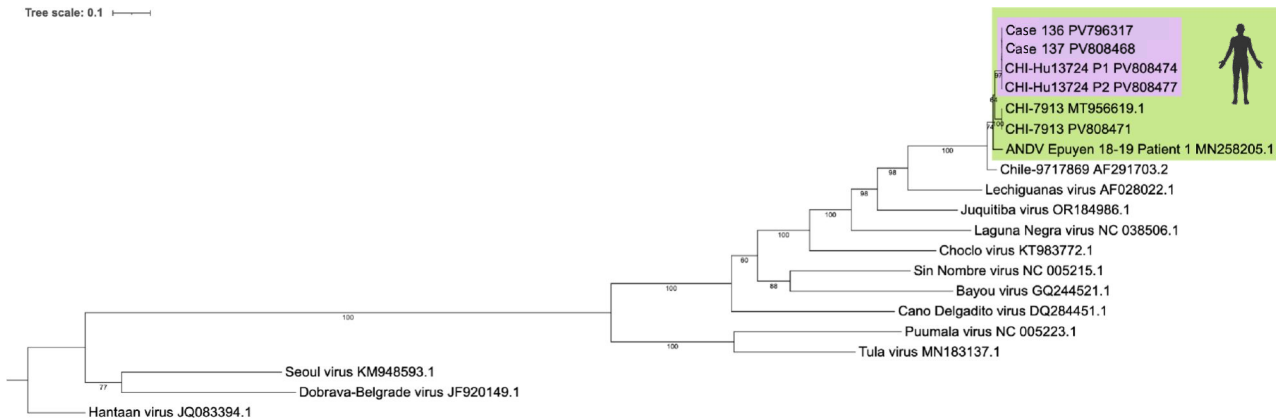

B

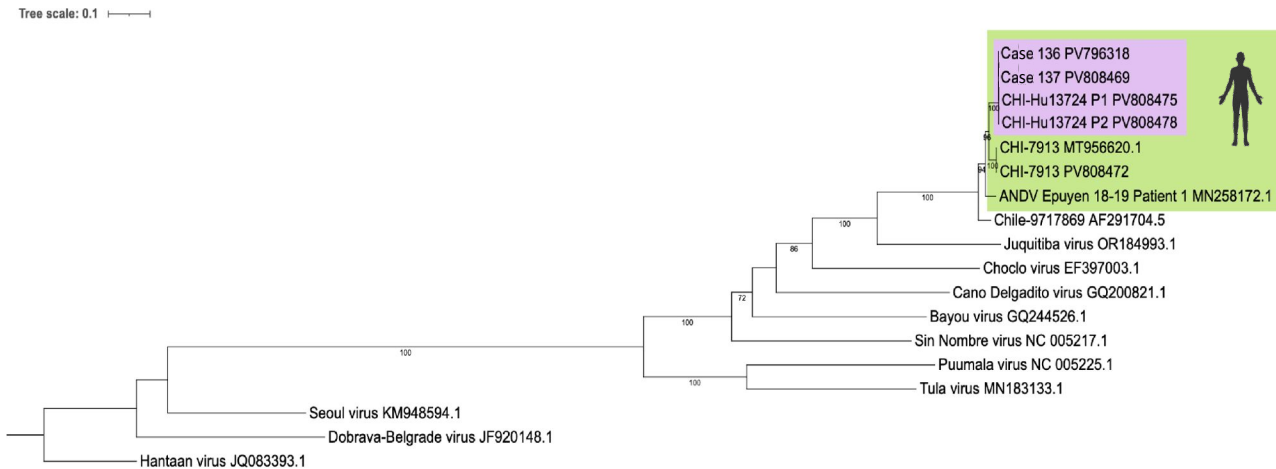

Supplement: Supplementary file 1 [file mmc1.zip › CRMS_Rev SuppFigure1.pdf]
